# The 2022 Lady Estelle Wolfson lectureship on neurofilaments

**DOI:** 10.1111/jnc.15682

**Published:** 2022-09-19

**Authors:** Axel Petzold

**Affiliations:** ^1^ Department of Neurodegeneration Queen Square Insitute of Neurology, UCL London UK

**Keywords:** biomarker, diagnosis, neurofilament, prognosis, treatment trial

## Abstract

Neurofilament proteins (Nf) have been validated and established as a reliable body fluid biomarker for neurodegenerative pathology. This review covers seven Nf isoforms, Nf light (NfL), two splicing variants of Nf medium (NfM), two splicing variants of Nf heavy (NfH), α‐internexin (INA) and peripherin (PRPH). The genetic and epigenetic aspects of Nf are discussed as relevant for neurodegenerative diseases and oncology. The comprehensive list of mutations for all Nf isoforms covers Amyotrophic Lateral Sclerosis, Charcot–Marie Tooth disease, Spinal muscular atrophy, Parkinson Disease and Lewy Body Dementia. Next, emphasis is given to the expanding field of post‐translational modifications (PTM) of the Nf amino acid residues. Protein structural aspects are reviewed alongside PTMs causing neurodegenerative pathology and human autoimmunity. Molecular visualisations of NF PTMs, assembly and stoichiometry make use of Alphafold2 modelling. The implications for Nf function on the cellular level and axonal transport are discussed. Neurofilament aggregate formation and proteolytic breakdown are reviewed as relevant for biomarker tests and disease. Likewise, Nf stoichiometry is reviewed with regard to in vitro experiments and as a compensatory mechanism in neurodegeneration. The review of Nf across a spectrum of 87 diseases from all parts of medicine is followed by a critical appraisal of 33 meta‐analyses on Nf body fluid levels. The review concludes with considerations for clinical trial design and an outlook for future research.

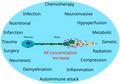

## Graphical abstract

Neurofilament proteins (Nf) are a validated bodyfluid biomarker for neurodegeneration. Nf are obligate, heteropolymers of different molecular sizes. Molecular assembly is hierarchical. Mutations and post‐translational modifications are extensive, relevant to pathology and immunotest development. The mature Nf proteins contain intrinsically unstructured polyampholytic regions relevant for protein–protein interactions. Contemporary state‐of‐the art laboratory tests quantify small, stable, soluble, proteolytic Nf breakdown products. Review and meta‐analyses of the clinical spectrum illustrate the relationship with neurodegeneration. Interpretation of increased human body fluid Nf concentration conveys information on the following: (i) diagnosis; (ii) prognosis; (iii) monitoring of progression and (iv) treatment trial endpoints.

**Abbreviations**
AD: Alzheimer disease
ADP: adenosine diphosphate
AE: acridinium ester
AF: atrial fibrillation
AGEs: advanced glycation end products
AIDS: acquired immunodeficiency syndrome
Ala: alanine
ALS: amyotrophic lateral sclerosis
AMD: age‐related macular degeneration
AQP4: aquaporin 4
Arg: arginine
Asn: asparagine
Asp: aspartate
ATTRm: hereditary transthyretin amyloidosis
BBB: blood–brain barrier
BMI: body mass index
BPAN: beta‐propeller protein‐associated neurodegeneration
Ca: calcium
CADASIL: cerebral autosomal dominant arteriopathy
CAG: cytosine‐adenine‐guanine
CBD: corticobasal degeneration
CHD: congenital heart disease
CI: confidence interval
CIDP: chronic inflammatory demyelinating polyneuropathy
CIN: critical illness neuropathy
CIPN: chemotherapy induced peripheral neuropathy
CIS: clinically isolated syndrome
Cit: citrullin
CJD: Creutzfeldt–Jakob Disease
CLN: Batten disease gene
CMT: Charcot Marie Tooth disease
CNS: central nervous system
COVID‐19: coronavirus disease 2019
CPB: cardiopulmonary bypass
CSF: cerebrospinal fluid
CTE: chronic traumatic encephalopathy
CV: coefficient of variation
Cys: cysteine
Da: dalton
DAT: dopamine transporter
DLB: diffuse lewy body dementia
DNA: deoxyribonucleic acid
ELISA: enzyme‐linked immunosorbent assay
FTLD: frontotemporal lobar dementia
GAN: giant axonal neuropathy
GBS: Guillain–Barré Syndrome
Glu: glutamine
Gly: glycine
GM: gangliosidosis
HD: Huntington's disease
HE: herpes encephalitis
HIV: human immunodeficiency virus infection
HMS: hypogravitatational motor syndrome
Hyl: hydroxylysine
Hyp: hydroxyproline
ICH: intracranial haemorrhage
IF: immunomagnetic reduction technology
Ile: isoleucine
INA: internexin alpha
IRT: immunomagnetic reduction technology
LBD: Lewy body disease
Leu: leucine
Lys: lysine
MAG: myelin‐associated glycoprotein
MCI: minimal cognitive impairment
MDMA: 3,4‐methylenedioxymethamphetamine
Met: methionine
MMN: multifocal motor neuropathy
MOGAD: myelin oligodendrocyte glycoprotein antibody disease
MS: multiple sclerosis
MSA: multisystem atrophy
Mw: molecular weight
Nf: neurofilament
NfH: neurofilament heavy chain
NfL: neurofilament light chain
NfM: neurofilament medium chain
nfvPPA: nonfluent and agrammatic variant primary progressive aphasia
NMOSD: neuromyelitis optica spectrum disease
ON: optic neuritis
OSAP: obstructive sleep apnoea syndrome
P: phosphorus
PAD: peptidyl arginine deiminases
PD: Parkinson disease
pI: isoelectric point
PML: progressive multifocal leukoencephalopathy
PNS: peripheral nervous system
PPA: primary progressive aphasia
Pro: proline
PRPH: peripherin
PSP: progressive supranuclear palsy
PTM: post‐translational modifications
RD: retinal detachment
RNA: ribonucleic acid
ROM: ratio of means
ROS: reactive oxygen species
SAH: subarachnoid haemorrhage
SAS: sleep apnoea syndrome
SCA: spinocerebellar ataxia
SDS: sodium dodecyl sulphate
Ser: serine
SIMOA: single‐molecule arrays
SMA: spinal muscular atrophy
SUMO: small ubiquitin‐related modifier
SVD: small vessel disease
svPPA: semantic variant Ppa
TBI: traumatic brain injury
Thr: threonine
Tyr: tyrosine
Val: valine
WNV: West Nile virus

## 1 INTRODUCTION

The Lady Estelle Wolfson Lectureship in Translational Medicine is awarded annually by the Royal College of Physicians in London to research with demonstrable patient benefit. The 2022 Lectureship was awarded to ‘Neurofilaments’ in acknowledgment of the successful efforts to deliver a reliable biomarker for neurodegeneration. As a consequence of worldwide, collaborative efforts, patients have now access to novel treatment strategies, the efficacy of which was elegantly demonstrated by implementing neurofilaments (Nf) as clinical trial outcome measures. The use of Nf permits to gain much quicker information on the progression of neurodegeneration compared to clinical metrics.

There is consensus that Nf have delivered the first biomarker for neurodegeneration which has, as a laboratory test, broken through the clinical specialty barrier. Industrial scale testing capabilities of Nf from blood samples have become possible by focusing on highly stable and soluble Nf polypeptides. A key development was the move from cerebrospinal fluid (CSF) to blood samples. The first reports on blood Nf concentrations appeared in 2004 (Khalil et al., 2018; Petzold, 2005b). Since, the field has been taken forward by a network approach between basic science, industry, laboratory and clinical science. Analytical sensitivity has been improved by employing Single‐Molecule Arrays (SIMOA) (Kuhle et al., 2016), Acridinium Ester (AE) technology (Center of Disease Control, 2022) and Immunomagnetic Reduction Technology (IRT) (Liu, Lin, et al., 2020). Novel chemical sensing platforms are at the brink of providing point‐of‐care tests (Chen, Tong, & Yang, 2021; Kim, Lee, & Park, 2020). Validation studies covered the performance of the test (Miller et al., 2016; Petzold, Altintas, et al., 2010) and the clinical relevance (Zetterberg & Blennow, 2018). The validation phase was rapidly followed by the successful implementation of Nf as a clinical trial endpoint (Hauser et al., 2020; Tabrizi et al., 2019).

This review will start with the genetics of the Nf proteins. This review expands from the strong focus on NfL in (Khalil et al., 2018) to include all seven known Nf isoforms. The reason for this will be motivated in the genetic section of this review which includes confirmed splicing variants. The section also includes an in‐depth update on known Nf mutations and related diseases. It will be learned that one important aspect of neurodegeneration is related to Nf aggregation. The susceptibility for Nf aggregates can further be increased by post‐translational modifications (PTM), notably phosphorylation, which will be reviewed in detail. Because of the poor solubility of Nf aggregates they do not make for a good body fluid biomarker. Therefore, the review continues with a discussion of described proteolytic breakdown products of Nf isoforms. This includes relevant relationships with disease. Finally, the clinical disease spectrum benefiting from Nf as a biomarker will be reviewed per medical specialty. The wealth of data on Nf has enabled meta‐analyses which will be reviewed in a separate section of the review for two reasons. First, they are likely to increase in number, are frequently cited and influence the interpretation of results. Second, there are meta‐analyses‐specific methodological points to be reviewed (Forgrave et al., 2019). The implications of these findings for clinical trials will be summarised.

## 2 METHODS

Google Scholar and PubMed were searched. This review refers to human Nf isoforms in general. This review includes Nf structural modelling using Alphafold2 through ColabFold (release version 2022/4/29) (Mirdita et al., 2021). Post‐translational modifications (PTM) were introduced to the Alphafold2 model with PyTMs (Warnecke et al., 2014). PBD files were processed in PyMOL (version 2.5.0) (Schrödinger, LLC, 2015) for creation of images. All scripts and models are available as Supplementary Data.

## 3 NEUROFILAMENTS: BUILDING BLOCKS FOR NEURONS AND AXONS

Nf are the key building blocks of the cytoskeleton for neurons and axons. Nf belong to the large family of intermediate filaments (IF) which have a diameter of ≈10 nm. This diameter is intermediate, hence the name, between microfilaments (≈7 nm) and microtubules (≈25 nm).

To truly admire the mechanistic achievement of Nf for cellular architecture one must put the size of neurons and axons into relation. For didactic reasons simplified, the neuronal cell bodies (≈10 μm size) are connected by axons over ≈1 m length (for the sciatic nerve). This is a factor of 100,000 difference. If St. Paul's cathedral (height 111.25 m) would represent a neuron, then the lengths of the axon would be ≈111,250 km long and ≈11 m wide. There would be no town on earth far enough away from London to, within this analogy, represent the next connecting neuron. The distance from London to Paris is 456 km, to New York 5570 km and to Sydney 17,000 km. The imaginary axon, originating from St. Paul's cathedral in London, would only stop about one‐third on the way to the moon. Such a structure would be impossible to build for men. And yet the function of Nf is not only to keep this structure stable, but also helps with the housekeeping.

## 4 FROM DNA TO PTM

**FIGURE 1:**
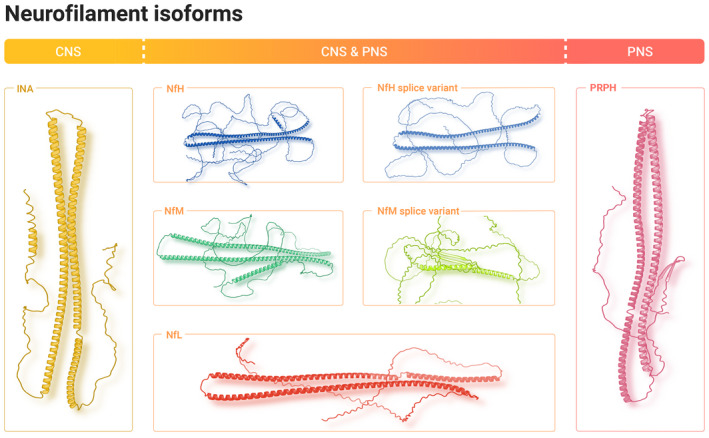
The seven human neurofilament isoforms. Structure models of the Nf isoforms are shown as a protein cartoon. The α‐helical domains are structured and contain a highly conserved rod domain common to all Nf isoforms. Expression is restricted to the CNS for INA (in yellow) and to the PNS for PRPH (in magenda). The remaining isoforms NfH (in blue), NfM (in green) and NfL (in red) are not specific for one single nervous system. NfL, NfM and NfH are expressed both in the CNS and PNS. Currently available immunoassays quantify NfL, NfH or NfM.

All Nf genes arose through gene duplication from an ancestral IF gene ≈800 million years ago (Lasek et al., 1985). Chromosome 8 encodes for Nf light (NfL) and Nf medium (NfM), chromosome 22 encodes for Nf heavy (NfH), chromosome 10 for internexin‐α (INA) and peripherin (PRPH) on chromosome 12. The genetic nomenclature has developed over the years with many synonyms, not all correct, used in the literature which may be found confusing. Therefore, an overview is given on the terms currently used (EMBL‐EBI, 2022; NIH, 2022). The structures of the Nf isoforms and splicing variants are shown in (Figure 1).

### 4.1 Nf identifiers and aliases

The order of the Nf isoforms is descending, according to the molecular weight (Table 1).

**TABLE 1:** The genetic background of Nf isoforms explains changes in reported weight due to alternative splicing for NfH and NfM

	NfH	NfM	NfL	INA	PRPH
Chromosome	22	8	8	10	12
Gene location	29,480,218–29,491,390	24,913,761–24,919,093	24,950,955–24,956,612	49,295,147–49,298,686	49,295,147–49,298,686
Length 1^a^	1020	916	543	499	470
Length 2^b^	924	540	−	−	−
Weight 1 (Mw)^c^	112477.56734 ± 7.23404	102470.81258 ± 6.54664	61400.80804 ± 3.95853	55389.99135 ± 3.54851	53650.26292 ± 3.46054
Weight 2 (kDa)^d^	105.6	102.5	61.5	55.4	53.7
Weight 3 (kDa)^e^	190–210	150	68	66	57
Charge^f^	−11	−64	−49	−14	−15
Phosphorylation	+++^g^	++	+	+	+
O‐glycosylation	++	++	+	−	−
Genetic risk for	ALS/SMA, CMT	ALS, PD	ALS, CMT	PD, LBD	ALS

*Note*: The weight increases following translation due to post‐translational modifications, the most important of which is phosphorylation. A number of mutations (see main text) have been associated with an increased risk for disease which is either autosomal dominant, autosomal recessive or considered a genetic susceptibility factor. Da, Dalton; Mw, molecular weight; pI, basal isoelectric point.

^a^ Full protein length.

^b^ Shorter lengths following alternative splicing.

^c^ Calculated from DNA sequence.

^d^ Reported from processed DNA sequence (EMBL‐EBI, 2022).

^e^ Migration in SDS gel which differs from the calculated weights because of post‐translational modification (PTM).

^f^ Calculated from amino acid sequence.

^g^ NfH is the most extensively phosphorylated protein of the human body.

#### 4.1.1 NfH

The gene ID for NfH is 4744, MIM 162230 (NIH, 2022) and P12036 (EMBL‐EBI, 2022). The location is on Chromosome 22 (29480218.0.29491390), NC. Aliases used are Neurofilament heavy polypeptide, NfH, NEFH, NfHHUMAN, CMT2CC, KIAA0845, NFH200 kDa neurofilament protein, Neurofilament triplet H protein.

#### 4.1.2 NfM

The gene ID for NfM is 4741, MIM 162250 (NIH, 2022) and P07197 (EMBL‐EBI, 2022). The location is on Chromosome 8 (24913761.0.24919093), NC. Aliases used are Neurofilament medium polypeptide, NfM, NEFM, NFMHUMAN, NEF3, NF‐M, 160 kDa neurofilament protein, Neurofilament triplet M protein, Neurofilament 3.

#### 4.1.3 NfL

The gene ID for NfL is 4747, MIM 162280 (NIH, 2022) and P07196 (EMBL‐EBI, 2022). The location is on Chromosome 8 (24950955.0.24956612), NC. Aliases used in the contemporary literature are as follows: Neurofilament light polypeptide, 68 kDa neurofilament protein, Neurofilament triplet L protein, NfL, NEFL, NFLHUMAN, CMT1F, CMT2E, CMTDIG, NF‐L, NF68, PPP1R110.

#### 4.1.4 INA

The gene ID for INA is 9118, MIM 605338 (NIH, 2022) and Q16352 (EMBL‐EBI, 2022). The location is on Chromosome 10 (103277138.0.103290346), NC. Aliases used are Alpha‐internexin, α‐internexin, Alpha‐Inx, INA, AINXHUMAN, FLJ18662, FLJ57501, NEF5, NF‐66, NF66, TXBP‐1, tax‐binding protein, neuronal intermediate filament protein alpha, 66 kDa neurofilament protein, Neurofilament‐66, Neurofilament 5.

#### 4.1.5 PRPH

The gene ID for peripherin is 5630, MIM 170710, PRPH and P41219 (EMBL‐EBI, 2022). The location is on Chromosome 12 (49295147.49298686), NC. Aliases used are Peripherin, NEF4, PERIHUMAN and Neurofilament 4.

### 4.2 Nf mutations

Pathologically relevant mutations of the Nf genes have been described mainly for ALS (NFH) and CMT (NFL). These mutations reviewed comprehensively here, expand substantially on previous reviews by the inclusion of synonymous variants which have been suggested to be also association with an increased genetic risk for disease and inclusion of protective variants (Table 1).

- ALS: NfL (E7K, Q93Q, I261I, I351V R241R, R421X, Y242Y,Y443Y, Y470S, G527del, GAG deletion 528E, Ter531G [Lin, et al., 2021]), NFM (S7W,F35Y, A144T, R295S, S279R, R311L, G407S, A475T, P499P, V718A, V755L, T831T, V858I [Lin, et al., 2021]), NfH (G35G, A90V, Q117X, R148P, E152D, E152D, A152V, Q171H, D187N, A203P, G249S, S285R, A314V, T338I, R346H, R352S, A380T, A380T, E463K, E491K, P505L, P5125, A528delGCT, V578V, P615L, T642M, P655deIAGA, E658 K665del, P663deITGAGAAGGCCAAGTCCCC, P663delTGAA744deIGCC, V670E, A672E, E673E, A674A, A708ins84bp, S787R, 2368–2370delAAG, K794K, E805A, P848S, K867N, E868K, T905I, E918G ([Lin, et al., 2021]), PRPH (A141T (Leung et al., 2006)). An intronic NfH (TTTA) variant (rs140814097) halved the risk for spinal onset ALS (Theunissen et al., 2022).
- CMT: NfL (P8L, P8Q, P8R, T21Afs*83, P22R, P22S, P22T, E90K, L94P, N98.S, N98T, E140*, A149V, E186*, E210, Y265C, L268P, L268R, Y265C, L268P, L268R, L311P, L312P, C322N326del, Q332P, L333P, Q334P, L336P, I384F, Y389C, E396K, G397L, R421*, F439I, P440L, Y443N, K467N (Butinar et al., 2008; Horga et al., 2017; Kim et al., 2022)). This list seems long, but overall only 27/798 (9%) of patients with CMT in a large Japanese cohort were due to a mutation in NFL (Higuchi & Takashima, 2022). NH (P739S, L1003A, A1004G, P1007A, P1008A, L1010G, L1015G, L1020G, L1020I (Ando et al., 2022; Pipis et al., 2021; Yan et al., 2020)). Only eight out of 2494 (0.003%) of patients with CMT in a large UK cohort were due to a mutation in the NfH gene making this an extremely rare condition (Pipis et al., 2021). All the NfH mutations in CMT affect the tail domain of the protein.
- SMA: NfH (P1007A in one single case (Ando et al., 2022))
- PD: NfM (S336G (Lavedan et al., 2002)). It was suggested that this might represent a PD susceptibility factor similar to NFM (P725G and deletion of valine in position 829) (Han et al., 2005; Krüger et al., 2003). INA (E46K) (Zarranz et al., 2003).
- LBD: INA (E46K) (Zarranz et al., 2003).

### 4.3 PTMs of Nf

**FIGURE 2:**
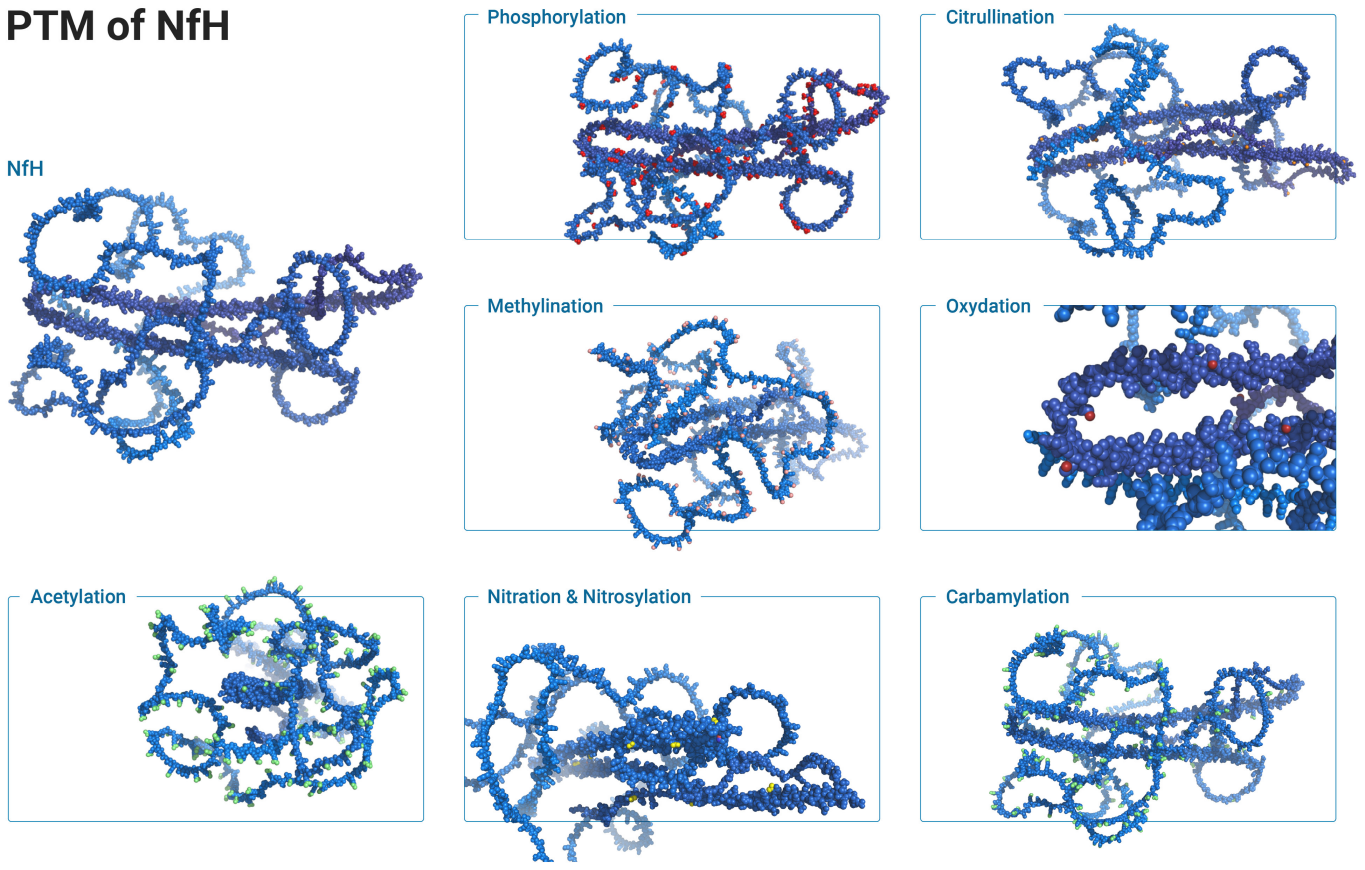
Post‐translational modifications of neurofilaments. The structure of NfH is represented as spheres in blue.  Residues modified by PTMs are coloured. The most abundant PTM is phosphorylation (red) at Ser, Thr and Tyr. There is extensive phosphorylation of the unstructured NfH tail domain. In contrast, citrullination (orange) is more extensive in the α‐helical domains. The 3D orientation of NfH is changed through this image which permits a better perception of the predominant effect of acetylation (green) and methylenation (salmon) to the unstructured domains. Data on oxygenation (dark red) are sparce and here shown in magnification for the residues where the two large α‐helices meet (far left). Likewise, nitration and nitrosylation are concentrated to the opposite end (far right) of the same α‐helices. Finally, carbamylation (green) is more expensive in the intrinsically unstructured domains. PTMs for the other Nf isoforms follow a similar pattern, but are less extensive than for NfH.

The transcription of Nf (Nf DNA → Nf mRNA) and translation (Nf mRNA → Nf amino acid sequence) is followed by PTMs (Figure 2). The most extensive PTM is Nf phosphorylation (Grant & Pant, 2000) (Table 1). The function of PTMs is to biochemically modify the ‘naked’ amino acid sequence (MacTaggart & Kashina, 2021). It has been anticipated that all reactive residues, at least at the protein surface, are subjected to PTMs of which only a fraction are currently known (MacTaggart & Kashina, 2021). PTMs regulate intermediate filament function (Snider & Omary, 2014). This entails the modification of axonal growth and myelination, synapse plasticity, neuronal differentiation, axonal diameter and transport, reaction to damage which includes autoimmunity. PTMs are also relevant to stability and protein interactions (Zecha et al., 2022). The list of known IF PTMs has increased from previous reviews (Khalil et al., 2018; Petzold, 2005b; Sihag et al., 2007).

#### 4.3.1 Nf phosphorylation & dephosphorylation

NfH is the most extensively phosphorylated protein of the human body. Phosphorylation of Nf isoforms occurs at three amino acids: Thr→Thr(P), Ser→Ser(P) and Tyr→Tyr(P) (see red dots in Figure 2) and is catalysed by kinases (Grant & Pant, 2000). Nf isoform phosphorylation affects protein charge (more negative) and molecular weight (heavier, see also Table 1). Phosphorylation is largely enzymatic and most abundant in the C‐terminal tail domain. The C‐terminal tail domain of NfH contains 42 KSP (Lys‐Ser‐Pro) repeats. Consequently, phosphorylation of the C‐terminal tail domain is, on the whole, regulated by proline‐directed kinases (Grant & Pant, 2000). The N‐terminal domain of the Nf isoforms is phosphorylated by a range of non‐proline‐directed enzymes: cyclin‐dependent kinase‐5, glycogen synthase kinase‐3 and extracellular signal‐regulated kinases (Grant & Pant, 2000; Petzold, 2005b).

Phosphorylation causes charge‐repulsion (Jones & Safinya, 2008). Charge‐repulsion modifies the axonal diameter. Increased Nf phosphorylation results in an increase in axonal diameter. Resistance to proteases also increases with phosphorylation (Goldstein et al., 1987), as does immunogenicity (Carden et al., 1985; Cloos & Christgau, 2004). Phosphorylated Nf isoforms remain immunogenic for millennia (Petzold et al., 2020). Phosphorylation affects Nf solubility and dynamics within the cytoskeleton which helps reorganisation of the Intermediate filament network (MacTaggart & Kashina, 2021). Nf phosphorylation increases with myelination during postnatal development (Shaw & Weber, 1982). Myelin‐associated glycoprotein (MAG) modifies phosphorylation of NfH and NfM (Dashiell et al., 2002). Abnormal Nf phosphorylation is observed in ALS and AD. The data for human NfH, NfM and NfL phosphorylation were recently summarised (MacTaggart & Kashina, 2021) and have been further updated (PhosphoSitePlus) and expanded here to include INA and PRPH (Hornbeck et al., 2015).

- NfH at amino acid residues: Ser54, Ser59, Ser61, Ser63, Tyr111, Tyr146, Ser337, Tyr385, Ser431, Thr501, Ser503, Ser511, Ser518, Ser526, Ser532, Ser540, Ser546, Ser552, Ser560, Ser566, Ser574, Ser580, Ser594, Ser600, Ser606, Ser614, Ser620, Ser628, Ser634, Ser640, Ser648, Ser654, Lys657, Ser660, Ser668, Ser674, Ser682, Ser688, Ser696, Ser702, Ser710, Ser716, Ser724, Ser730, Thr738, Ser744, Ser752, Ser758, Ser769, Ser782, Ser793, Ser801, Ser807, Ser828, Thr911, Ser948
- NfM at amino acid residues: Ser23, Ser28, Ser30, Ser33, Ser37, Ser44, Ser55, Ser73, Ser226, Ser333, Tyr384, Tyr401, Ser467, Ser511, Ser545, Ser553, Ser558, Ser559, Ser615, Ser620, Ser628, Ser633, Ser641, Ser646, Ser654, Ser659, Ser667, Ser670, Ser672, Ser680, Ser685, Ser736, Thr748, Thr750, Ser783, Ser788, Ser821, Ser837, Tyr872
- NfL at amino acid residues: Ser3, Tyr14, Thr21, Ser56, Ser103, Thr154, Ser215, Ser221, Tyr372, Tyr389, Ser472, Thr520
- INA at amino acid residues: Ser23, Ser30, Ser58, Ser78, Tyr272, Tyr287, Ser335, Tyr379, Tyr396, Thr442, Ser445, Thr463, Ser464, Ser496
- PRPH at amino acid residues: Ser13, Tyr17, Ser28, Ser50, Ser59, Ser62, Ser75, Tyr287, Ser405, Ser413, Thr421

#### 4.3.2 Nf citrullination

Citrullination (synonymous: peptidylarginine deimination or just deamination) alters peptidyl‐Arginine, but not free Arginine, to peptidyl‐Citrulline (Arg→Cit). This is achieved through Ca^2+^‐dependent hydrolysis of ketamine to keton (Mondal & Thompson, 2021). The reaction is catalysed by a group of peptidyl arginine deiminases (PADs). Citrullination reduces charge (−1 per deimination), weight (+0.98 mW), susceptibility to proteolysis, structure, function, neurodegeneration and autoimmunity (Briot et al., 2020). Given the amount of data on citrullination of human MBP (Cao et al., 1998; Srour et al., 2022; Wang, Chen, et al., 2022; Yu & Proost, 2022), which has only 29 Arg residues, it is surprising how little is known regarding the citrullination of human NfH (44 Arg), NfM (37 Arg), NfL (35 Arg), INA (45 Arg), PRPH (48 Arg). Citrullination in Figure 2 was modelled in orange spheres. Analytical limitations for experimental study of citrullination (Verheul et al., 2018) have been overcome with high‐resolution mass spectrometry (Yu & Proost, 2022).

#### 4.3.3 Nf glycosylation

Glycosylation reversibly adds carbohydrates. Glycosylation has been demonstrated for NfL, NfM and NfH (Dong et al., 1993). In humans, there is O‐ and N‐glycosylation. N‐glycosylation targets Asn. O‐glycosylation (O‐GlcNAcylation) occurs at the hydroxyl‐group of Thr, Ser, Hyl and Hyp: Asp→Asp‐glycan, Ser→Ser‐glycan. Glycosylation is central to a range of immune processes. Altered protein glycosylation may trigger an autoimmunity (Cloos & Christgau, 2004). Impaired glycosylation has been related to neurodegeneration (Yuzwa et al., 2012). Reduced O‐GlcNAc glycosylation of NfM is linked to AD and ALS (Deng et al., 2007; Dong et al., 1996). Glycosylation of NfH increased with glucose deprivation (Cheung & Hart, 2008).

#### 4.3.4 Nf glycation

Glycation describes the non‐enzymatic addition of a sugar aldehyde or ketone to an amino acid residue (Kikuchi, 2003). This is the main difference to glycosylation which requires enzymes, but the terms are not always strictly differentiated in the literature. Glycation is rapid for surface Lys residues, particularly if adjacent to His. The late stage of glycation leads to formation of advanced glycation end products (AGEs) which are cytotoxic. Many AGEs are unstable and some are immunogenic (Chou et al., 1998; Cloos & Christgau, 2004; Virella et al., 2003). There is a likely synergistic role of glycation and oxidative stress regarding neurotoxicity in AD and ALS (Kikuchi, 2003).

#### 4.3.5 Nf acetylation

Acetylation of Lys and Arg (both basic) neutralises the side chain resulting in a larger residue with reduced polarity (green coloured in Figure 2) (Lacoursiere et al., 2022). There are at least 40 lysine acetyltransferases (Donev, 2021) and ≈20 deacetylases (Ho et al., 2020). Acetylation in human Nf has been demonstrated for (MacTaggart & Kashina, 2021):

- NfH at amino acid residues: Lys659, Lys663, Lys907, Lys908
- NfM at amino acid residues: Lys298, Lys403, Lys653, Lys693, Lys698
- NfL at amino acid residues: Lys362, Lys379, Lys391
- INA at amino acid residues: Lys290, Lys398, Lys483, Lys498
- PRPH at amino acid residues: Lys288, Lys398

#### 4.3.6 Nf deamidation

Deamidation has been described as a molecular clock because it is closely related to the physiological half life of a protein (Ying & Li, 2022). The lifetime of Nf isoforms was calculated to be in the range of 1–2 years (Lee & Cleveland, 1996). Spontaneous deamination mostly affects Asn→Asp and is very slow for Gln→Glu. Enzymatic deamidation is catalysed by tissue transglutaminase in a Ca^2+^‐dependent manner. With high‐resolution mass spectrometry it has become easier to quantify deamidation (mass shift of 0.984 Da) (Ying & Li, 2022). Deamidation has been described in AD, PD among other neurodegenerative conditions (Briot et al., 2020). Therefore, it is interesting to note that yet so little is known about deamidation of Nf isoforms (see supplementary material in (Trimpin et al., 2004)). Deamination is interrelated with isomerisation and racemisation.

#### 4.3.7 Nf isomerisation

Isomerisation describes the conversion of for example Asp→isoAsp or GLy→isoGly. Isomerisation almost always affects susceptibility to proteolysis. Protein isomerisation has been implicated in ALS pathogenesis (Parakh et al., 2013).

#### 4.3.8 Nf racemisation

Asp→D‐Asp (D‐isoAsp), Glx→DGlu (D‐isoGlu). Occasionally also other residues such as Ala, Ser, Thr may racemise. Susceptibility to proteolysis is always affected. There are yet no systematic data on racemisation and isomerisation on human Nf isoforms.

#### 4.3.9 Nf oxidation

Oxidative stress causes damage to proteins which is relevant in neurodegenerative disease (Lee et al., 2021). Even in absence of pathology, there will be oxidative stress to any given protein over time. One major target of oxidation is the thiol side‐chain of Cys (dark red spheres in Figure 2) (Lee et al., 2021). Given the longevity of Nf isoforms, it is not surprising that oxidation has been described (Trimpin et al., 2004). Oxidising compounds such as free radicals are commonly known as reactive oxygen species (ROS) and the major cellular sources are mitochondria. It has been proposed that NfH protects other axonal proteins by accumulating the oxidative damage (Couillard‐Despres et al., 1998).

#### 4.3.10 Nf S‐nitrosylation

Similar to oxidation, S‐nitrosylation (S‐nitrosation) also affects the thiol side‐chain of Cys. A good pre‐analytical quality control pipeline is relevant to avoid artefacts (Wang, Zhou, et al., 2022). Modelling of nitrosylation and nitration is shown in yellow colour in Figure 2.

#### 4.3.11 Nf methylation

The methylation of Lys and Arg residues is catalysed by methyltransferases and reversed by demethylases. Both amino acids can be mono‐ and dimethylated, with trimethylation also being possible for Lys. Methylation of Lys is illustrated in Figure 2 (salmon colour), but there are no experimental data yet confirming that this happens in vivo. Contemporary experimental data provide evidence for methylation of Arg in Nf isoforms (Ho et al., 2020; MacTaggart & Kashina, 2021):

- NfH at amino acid residues: Arg164, Arg412, Arg389
- NfM at amino acid residues: Arg21, Arg26, Arg42, Arg54, Lys102, Arg111. Arg382
- NfL at amino acid residues: Arg23, Arg30, Arg37
- INA at amino acid residues: Arg24, Arg39, Arg104, Arg377
- PRPH at amino acid residues: Arg72, Lys98

#### 4.3.12 N‐terminal Nf modifications

In humans, the N‐terminus is frequently N‐acetyl ‘blocked’ and common residues are as follows: Ala, Ser, Met, Gly or Thr. Enzymatic removal of these residues is possible. N‐terminal acetylation has been shown for all Nf isoforms (Trimpin et al., 2004).

#### 4.3.13 C‐terminal Nf modifications

Amidation of the C‐terminus is common. Gly is a frequent donor for the amide. Other mechanisms are methylation and isoprenylation for GPI anchors and ADP‐dependent ribosylation of C‐terminal Lys (Dutour‐Provenzano & Etienne‐Manneville, 2021; Srour et al., 2022; Ying & Li, 2022).

#### 4.3.14 Nf ubiquitination

Ubiquitination largely affects lysine residues (Lacoursiere et al., 2022). This is catalysed by three enzymes, E1 (activates), E2 (conjugates) and E3 (ligates) ubiquitin (8.5 kDa) onto the Lys within Nf. Ubiquitination for Nf isoforms has been demonstrated for (MacTaggart & Kashina, 2021):

- NfH at amino acid residues: Lys31, Lys336, Lys531, Lys565, Lys659, Lys895
- NfM at amino acid residues: Lys118, Lys259, Lys263, Lys271, Lys445, Lys451, Lys871, Lys875
- NfL at amino acid residues: Lys15, Lys157, Lys271, Lys370
- INA at amino acid residues: Lys95, Lys290
- PRPH at amino acid residues: Lys288, Lys290, Lys398, Lys402

#### 4.3.15 Nf SUMOylation

The Lys residues of the Nf isoforms are a target for the covalent addition of a small ubiquitin‐related modifier (SUMO). This is catalysed by SUMO‐specific ligases and reversed by a family of SUMO/Sentrin‐specific proteases (Chen, Zhang, et al., 2021). Deficient SUMOylation results in protein accumulation, aggregation, alteration of synapses and ion channels (Henley et al., 2020; Moon et al., 2021). SUMOylation affects PRPH at Lys398 (Hornbeck et al., 2015).

#### 4.3.16 Nf Farnesylation

Farnesylation adds a lipid group to Cys residues which increases hydrophobicity. Farnesylation can be permanent or reversible and is modified by farnesyltransferase inhibitors (Young et al., 2013).

## 5 NF HETEROPOLYMER FORMATION

**FIGURE 3:**
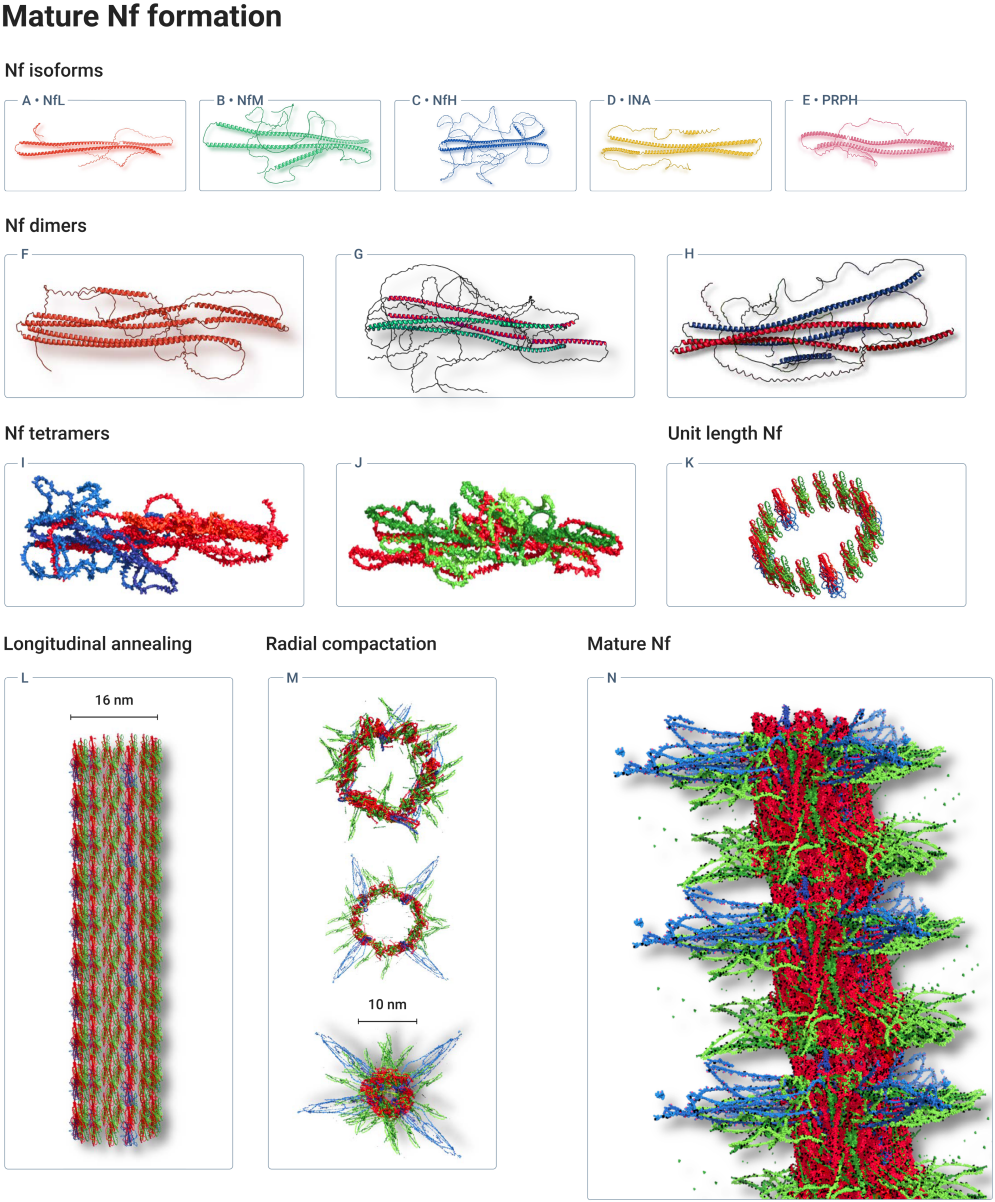
Neurofilaments are key building blocks of the neuro‐axonal cytoskeleton. The Nf heteropolymer is formed by parallel alignment of Nf isoforms to dimers, followed by antiparallel alignment of dimers to tetramers. Tetrameters assemble to a unit length filament. Through longitudinal annealing a ≈ 16 nm filament assembles which is finally radially compacted to the major ≈10 nm neurofilament. Structure models of the Nf isoforms are shown as a protein cartoon for (a) NfL, (b) NfM, (c) NfH, (d) INA and (e) PRPH. Dimer formation requires the presence of NfL and are shown here including visualisation of sidearm chains targeted by PTMs for (f) NfL:NfL, (g) NfL:NfM and (h) NfL:NfH. The interaction between the sidearms becomes more complex with formation of tetramers here shown as surface plots for (i) NfL:NfL with NfL:NfH and (j) for NfL:NfM with NfL:NfM. (k) Shows a single unit length Nf filament which is built from 16 tetramers (4 × (i) and 12 × (j)). (l) Longitudinal annealing of the unit length Nf filaments elongates the filament by 60 nm steps with each unit length added. (m) Radial compaction (sagittal view) of the 16 nm diameter of the unit length Nf to the final diameter of 10 nm (=10.2 Å measurement in pymol). During radial compaction, PTMs support the structural changes of the Nf side‐arms (coloured as in Figure 2). There is progressive emergence of Nf isoform side‐arms from the centre, radiating to the periphery. (n) Mature Nf.

Once expressed and modified through PTMs, the Nf isoforms co‐assemble to form a very stable threadlike heteropolymer (Figure 3). The process of the assembly is understood to require only a small central section within each of the Nf isoforms. This central section has a preserved structure. Simplified, Nf hetero‐polymerisation can be visualised as a 10–15 nm tight knot tied with the central part with two loose, flexible ends of the thread extending another 50–100 nm (represented as ribbons in Figure 3a–e) (Janmey et al., 2003). The C‐ and N‐terminal regions contain intrinsically unstructured domains which are governed by electrostatic forces (Ghosh et al., 2022). These electrostatic forces are responsible for the outward radiation of the charged, C‐terminal Nf tail regions from the core of the assembled Nf heteropolymer (Figure 3l–m). On a microscopic level, this results in charge‐repulsion and change of axonal diameter.

The Nf rod domain is subdivided into four helical domains: 1A, 1b, 2A, 2B. The helical domains are separated by linkers: L1, L12, L2. The 1B domain is flanked by an A11 mode of interaction pocket (N‐terminal) and A11 mode of interaction knob (C‐terminal). This knob‐pocket interaction is the key for alignment of Nf dimers (Figure 3f–h) to tetramers (Figure 3i, j) in phase with the hydrophobic 1B coil domain (Eldirany et al., 2021). Polymer stability mostly comes from the A11 knob‐pocket interactions. The Nf tetramers generally consist of NfL together with one of the other Nf isoforms. The need for one NfL per unit is explained by in vitro *data* showing that of the human Nf isoforms only NfL can self‐assemble in vitro (Carter et al., 1998). The heteropolymer is formed through co‐assembly of NfL, NfM and NfH (Yuan & Nixon, 2021). INA is added in the brain and PRPH in the peripheral nerve.

## 6 FUNCTION

Initially, it was proposed that Nf would only affect an organism indirectly through the structure and function of neurons (Lasek et al., 1985). This view is, in its core, still correct but has been expanded:

- Action potential conduction speed through axonal diameter (Lawson et al., 1993)
- Aldosterone secretion (Maniero et al., 2017)
- Axonal diameter (Jones & Safinya, 2008; Lasek et al., 1983; Lasek et al., 1985; Marszalek et al., 1996; Monaco et al., 1989)
- Axonal flow and stasis (Balaratnasingam et al., 2007; Vial, 1958)
- Axonal transport (Lasek, 1967; Mutalik & Ghose, 2020; Nixon & Logvinenko, 1986)
- Epigenetic regulation in cancer (Calmon et al., 2015; Hasan et al., 2021; Li et al., 2020)
- Evolution of the nervous system across species (Lasek et al., 1985)
- Interaction with mitochondria (Gentil et al., 2011; Wagner et al., 2003; Zhu et al., 2022)
- Interaction with myelin proteins (Dashiell et al., 2002)
- Mechanical stability (Kim et al., 2011)
- Modulation of the endoplasmic reticulum (Rao et al., 2011)
- Ontogeny (Pachter & Liem, 1984; Shaw & Weber, 1982)
- Synaptic modeling and startle response through activity at the presynaptic terminal (Bullock & Horridge, 1965)
- Viscoelastic properties (Herrmann et al., 2007; Srinivasan & Kumar, 2012)

## 7 AGGREGATION

Nf are prone to aggregation (Eldirany et al., 2021). The concept that Nf aggregation is relevant for disease originates in experimental studies on ALS (Julien, 2001). Nf aggregation causes slowing and disruption of axonal transport (Collard et al., 1995) which is followed by loss of neuronal function and disease (Didonna & Opal, 2019; Julien, 2001; Rebelo et al., 2016). Consistent with the experimental data, there is evidence for Nf aggregates in the blood of subjects with ALS (Adiutori et al., 2018). Future studies on Nf aggregation should consider the possibility that a limited degree of aggregate formation is physiological and reversible (Murray et al., 2022). The alignment of aggregation‐prone domains is facilitated by a steric zipper region and their side‐chain interdigitation. The reversibility of this limited aggregate formation is governed by mutations and PTMs (Murray et al., 2022). Irreversibility is increased by mutations introducing Cys, amyloidogenic elements and PTMs such as hyper‐phosphorylation (Murray et al., 2022; Rebelo et al., 2016; Xiao et al., 2006). It has been proposed that Nf aggregate formation is also a relevant factor for long‐term protein preservation (Petzold et al., 2020). Extremely tight folding is mechanically possible because of the large proportion of Lys, and intrinsically unstructured domains.

## 8 NF ISOFORM CLEAVAGE AND STABILITY

Pioneering experimental work on enzymatic cleavage of intermediate filaments used six enzymes enabling amino acid sequencing as a means of characterisation of the proteolytic breakdown products (Geisler & Weber, 1981). The enzymes employed were 2‐nitro‐5‐thiocyanobenzoic acid, trypsin, thermolysin, chymotrypsin, Staphylococcus aureus V8 protease and carboxypeptidase. In a second step, major Nf fragments were subjected to double chemical cleavage with NTCB and BNPS‐skatole (Geisler et al., 1982). This approach revealed a highly specific 5 K NfL cleavage product (Geisler et al., 1982). The amino acid sequence of this 5 K NfL cleavage product was as follows: *Arg‐Ala‐Ala‐Lys‐Asp‐Glu‐Val‐Ser‐Glu‐Ser‐Arg‐Arg‐Leu‐Leu‐Lys‐Ala‐Lys‐Thr‐Leu‐Glu‐Ile‐Glu‐Ala‐Cys* (Geisler et al., 1982). Further experiments showed that digestion of NfH revealed fragments of high stability which resisted prolonged treatment with enzymes (Autiliogambetti et al., 1986). The explanation for the different stability of the Nf isoforms to enzymatic digestion was through phosphorylation (Sternberger & Sternberger, 1983). Because NfH is more heavily phosphorylated than NfL it was found to be the more stable Nf isoform (Goldstein et al., 1987).

For clinical biomarker research stability is important. A stable biomarker can more readily be processed in routine laboratory practice. In contrast, a biomarker which is not stable and degrades rapidly requires fast and careful processing an immediate storage at −80°C (Teunissen et al., 2009). In this context, it needs to be acknowledged that early experimental studies (Autiliogambetti et al., 1986; Geisler et al., 1982; Sternberger & Sternberger, 1983) led to an insightful scientific exchange on Nf biomarker stability (Gunnarsson et al., 2011; Koel‐Simmelink et al., 2011). The data used for the two sides of the argument were, at the time, based on the assumption that the tests used quantified the full‐length NfL and NfH proteins. Both Nf isoforms could be shown in fresh blood and CSF samples, but the NfL signal was diminished in blood and abolished in CSF after storage of only 18 h at room temperature. The findings from the immunoblot were consistent with data from ELISA (Koel‐Simmelink et al., 2011). There was a decrease in the concentration of the full‐length NfL within 24 h which progressed to non‐measurable levels within 4 days. In contrast, full length, phosphorylated NfH persisted for up to 290 weeks (Koel‐Simmelink et al., 2011). These experiments were consistent with what was expected from the earlier literature (Autiliogambetti et al., 1986; Geisler & Weber, 1981; Goldstein et al., 1987; Sternberger & Sternberger, 1983).

How could the finding of higher NfL stability using a different ELISA (Gunnarsson et al., 2011) be explained? It was the serendipitous finding of very long‐term stability of NfL from an ancient human brain (Petzold et al., 2020) which stimulated re‐visitation of the question in a joint experiment (Altmann et al., 2021). This included the two groups involved in the scientific letter exchange on NfL stability (Gunnarsson et al., 2011; Koel‐Simmelink et al., 2011). The conclusion from these experiments is that the discussion has moved from stability of the full‐length protein to stability of protein fragments, likely to arise with pathology. The presumed NfL fragment currently quantified is of sufficient stability to be useful for routine laboratory sample handling, transport and storage (Altmann et al., 2021). The data confirm the 7‐day stability of NfL as a biomarker within a 4.4–5.5 pg/ml 95% CI in a Bland–Altman plot (Altmann et al., 2021). Western blot data (Figure S3, Brureau et al., 2017) revealed the absence of full‐length NfL from the CSF sample, but the capture of NfL fragments which is consistent with the Western blotting shown by (Goldstein et al., 1987; Koel‐Simmelink et al., 2011; Sternberger & Sternberger, 1983).

Notably, there has been progress on further characterisation of Nf cleavage products (Geisler et al., 1982). For NfL, it was shown that the full‐length protein was absent from Western blot (Brureau et al., 2017). Instead, a NfL‐specific cleavage product was found around 10 kDa (Brureau et al., 2017). The detailed supplementary materials also show binding of the monoclonal antibody used in (Gunnarsson et al., 2011) to other NfL breakdown products around 50, 40 and 20 kDa which were not specific for NfL because mass spectroscopy showed sequence overlap with Vimetin (P20152) and Desmin (P31001) (Brureau et al., 2017). The elegant methodological approach chosen was to use the commercially available ELISA coated with the capture antibody 47:3 (Figure 2, lane 8 Norgren et al., 2002) to collect protein from CSF which then were subjected to nanoLC Mass spectrometry analysis (Brureau et al., 2017).

These observations are consistent with data demonstrating that calpain mediates cleavage of axonal Nf during Wallerian degeneration (Ma et al., 2013). Proteolytic breakdown fragments for NfL were found around 22, 40 and 55 kDa. Of these, the ≈55 kDa fragment was least stable (Ma et al., 2013). The ≈53 and 57 kDa proteolytic NfL fragments have been demonstrated following traumatic brain injury (TBI) in vivo and in vitro (Nixon & Sihag, 1991; Posmantur et al., 1994; Posmantur et al., 1998). In addition, a 30 kDa NfL proteolytic fragment was described in blood samples (Adiutori et al., 2018). A combined immunoprecipitation and mass spectroscopy analysis of NfL cleavage products permitted to characterises NfL based on antibody‐targeted selectivity of head, rod and tail domains (Budelier et al., 2022). Data from brain and CSF samples were compared. The full‐length NfL protein was present in the brain only. The brain also contained a C‐terminal fragment (NfL_530−540_). In the blood, three NfL fragments dominated, NfL_92−224_, NfL_324−360_, NfL_530−540_. The latter was best for separating samples from individuals with controls. The supplementary data to this report provide a detailed list of the antibody and mass spectroscopy data which show that there are many more NfL fragments in the CSF (Budelier et al., 2022).

In TBI, proteolytic breakdown products for NfH have been described in a human brain tissue microdialysis study from the interstitial fluid (Petzold, Tisdall, et al., 2011). Cleavage was due to activation of an axonal membrane‐bound Ser protease. The enzyme cleaved NfH into the fragments NfH_476−986_ (56 kDa), NfH_476−1026_ (60 kDa), NfH_1−476_ (53 kDa), NfH_835−1026_ (21 kDa) and NfH_852−986_ (15 kDa). These in vivo characterised NfH fragments were different to earlier reported calpain cleavage products at 120 and 146 kDa (Greenwood et al., 1993).

Taken together, it is likely that there are many Nf isoform cleavage products which, because of their stability and solubility, are of advantage for use as a biomarker in clinical studies compared to the full‐length Nf proteins.

## 9 STOICHIOMETRY

Stoichiometry describes the quantitative relationship of several substances in a defined compartment. The stoichiometry of Nf proteins is noted as an averaged value for NfL:NfM:NfH in the following reports:

- 4:2:1 according to post‐mortem data from bovine spinal cords (Scott et al., 1985)
- 7:3:2 in mature axons (Janmey et al., 2003)
- 24:5:2 according to CSF data and Monte Carlo simulations in multiple system atrophy (Kim et al., 2011)
- 16:11:4 according to CSF data and Monte Carlo simulations in relapsing–remitting multiple sclerosis (Kim et al., 2011)
- 24:2.4:1.6 in the plasma of individuals with ALS (Zucchi et al., 2018)

These data suggest that, on an averaged group level, there is a change in Nf stoichiometry in disease. But the observation is contested (Yuan & Nixon, 2021) because it was not possible to demonstrate an isolated up‐regulation of a single Nf isoform gene (Robinson et al., 1994; Wong et al., 2000) or protein expression (Ashton et al., 2019) in ALS and AD. Limitations of these studies were, however, the artefacts due to the post‐mortem interval and averaging of data over a large histological area. This criticism aside, indeed selective suppression of NfL mRNA (p0.05) was shown in the lateral horn of post‐mortem cases with ALS if compared to controls (Wong et al., 2000). Therefore, Wong et al. concluded that the stoichiometry of IF expression is markedly disrupted, but cautioned against interpreting this as an ‘abortive regenerative response’. In another study, selective perturbation of NfH miRNAs is reported (Maciotta et al., 2013). Future studies will need to move to single cell level.

In vitro, hydrogel experiments provide further evidence that the Nf heteropolymer remains stable across a large range of Nf stoichiometries (Beck et al., 2012). Consistent with other studies, the ground truth was defined as NfL:NfM:NfH = 7:3:2 (; Beck et al., 2012; Janmey et al., 2003; Kim et al., 2011; Zucchi et al., 2018). The range of NfL:NfM:NfH stoichiometries studied in hydrogels were: 7:3:2; 80:20:0; 82:0:18; 90:10:0; 90:20:0; 92:0:8; 92:0:8; 97:0:3; 97:3:0; 100:0:0. These stoichiometries were tested at different salt concentrations: 48, 96 and 240 mM (Beck, Deek, Choi, et al., 2010). These hydrogel‐based findings (Beck, Deek, Choi, et al., 2010; Beck, Deek, Jones, & Safinya, 2010) reveal a relationship between Nf heteropolymer stiffness which depends on stoichiometry and salt concentration. An increase in NfL or NfM, but not NfH, in the stoichiometry results in a stiffer Nf heteropolymer. It was proposed that there is a stoichiometric governed competition between entropic Nf sidearm repulsion and electrostatic ionic bridging (Kumar et al., 2002). The experimental model is well suited for studying the effect of Nf PTMs changing sidearm charge reviewed here (Table 2, Figure 2). The need to fill this gap in knowledge on Nf has been highlighted as a research priority for informing future computational approaches and clinical studies (Khan et al., 2020; Zucchi et al., 2020).

**TABLE 2:** Post‐translational modifications (PTM) and their main target amino acid residue. The reversal of these PTMs are not included in the table and are discussed in the main text as relevant for Nf isoforms, for example Nf dephosphorylation to reverse Nf phosphorylation

PTM	Mechanism
ADP‐ribosylation	Adds ADP‐ribose which is a form of glycosylation. Affected are Gglu, Asp, sSr, Arg, Cys, Lys, diphthamide, phosphoserine, Asn
Acetylation	Acetylates Lys
C‐terminal	Amination (adds an amine group), glycosyl phosphatidylinositol attachment, phosphorylation
Carbonylation	Adds carbon monoxide
Carboxylation	Adds a carboxyl group to glutamate
Citrullination	Synonymous to deimination, converts Argto Cit
Farnesylation	Adds a lipid group
Glutamylation	Adds glutamate
Glycation	Adds a sugar aldehyde or ketone, the late stage is characterised by formation of advanced glycation end products (AGEs)
Glycosylation	Similar to glycation only that this is enzyme mediated
Glycylation	Adds glycine
Hydroxylation	Changes Pro to Hyp, also works with Lys
Isomerisation	Transformation into an isomer which changes the chemical structure
Methylation	Add a methyl group to Arg
N‐terminal	Arginylation (adds Argi), formylation (adds a formyl group), pyroglutamate (N‐terminal Glu forms a pyroglutamate group)
Nitrosylation	Adds nitric oxide
Oxidation	Adds oxygen species
Phosphorylation	Adds phosphorus to Ser and to a lesser degree also to Thr and Tyr
SUMOylation	Adds a small ubiquitin‐related modifier (SUMO)
Sulphation	Adds sulphate to Tyr
Tyrosination	Adds Tyr
Ubiquitination	Adds ubiquitin to Lys

Another limitation is that the knowledge on Nf stoichiometry is based on averaged data. Figure 3 illustrates that the unit length filament is constructed by 16 tetramers. This calculates to 64 individual Nf isoforms. It is likely that the Nf isoform stoichiometry varies between within the unit length and over the lengths of the longitudinally annealed filament.

For the treatment of neurodegeneration, the observations on Nf stoichiometry could be relevant. It was proposed that an adaptive Nf stoichiometry represents an endogenous mechanism to slow down the progression of neurodegeneration (Zucchi et al., 2018). A switch from the more resource demanding larger proteins, NfH and NfM, to a higher concentration of the smaller NfL in the heteropolymer comes at practically no risk to Nf heteropolymer stability (Kim et al., 2011), but helps the motor neuron to save time and energy (ADP) (Zucchi et al., 2018). Modulation of Nf stoichiometry should be of interest for gene silencing methods (McCampbell et al., 2018; Miller et al., 2020). The idea is to transiently and reversibly lower the expression of individual Nf isoforms. Such experiments should test if a gently applied gene silencing treatment approach can support an endogenous strategy of adaptive protein stoichiometry. Does the gentle down‐regulation of NfM and NfH prolong survival in motor neuron disease? And if so, can this be of benefit for treatment of other neurodegenerative conditions? Or use of Nf aggregation inhibitors.

## 10 QUANTIFICATION

The impressive advance from first‐generation immuno‐assays to fourth‐generation immuno‐assays had been subject to reviews with a strong focus on the quantification of NfL with SIMOA (Khalil et al., 2018; Li & Mielke, 2019). The main advantage of the, still very expensive, SIMOA approach is an increased detection limit and analytical range if compared to the relative cost‐effective, in house, enzyme‐linked immunosorbent assay (ELISA) (Gaetani et al., 2018; Norgren et al., 2003; Petzold et al., 2003; Rosengren et al., 1996; Shaw et al., 2005; Van Geel et al., 2005). The reported detection limits are 0.18 fg/ml for the immunomagnetic reduction (IMR) assay, 0.241–0.62 pg/ml for the SIMOA assay, 2.70 pg/ml for the Ella assay, 15.6 pg/ml for the ECL assay and 78.0 pg/ml for the ELISA (Gauthier et al., 2021; Kuhle et al., 2016; Liu, Lin, et al., 2020). A lower limit of quantification of 3.9 pg/ml has been advertised for Acridinium Ester (AE) technology (Center of Disease Control, 2022).

Head‐to‐head method comparisons demonstrate general agreement on pathological compared to normal NfL concentrations (Gauthier et al., 2021) or NfH concentrations (Petzold & Shaw, 2007), with a clear point for the higher sensitivity of the NfL SIMOA platform if compared to the NfL ELISA or an NfL electrochemiluminescence immunoassay (Kuhle et al., 2016). A limitation of some comparisons is that the protein standard curve used in the assays was not of identical origin (Gauthier et al., 2021; Kuhle et al., 2016). This has two implications; first test comparisons regarding the lower limit of detection are only indirect; second, it complicates the interpretation of Bland–Altman plots which are interpreted to show that the SIMOA assay underestimates lower range NfL concentrations by about 17% (Gauthier et al., 2021). Another high‐throughput immunoassay has been developed by Siemens Healthineers (Uzgiris et al., 2022). To address these points and downstream effects experts at the interface between pharmacy and regulatory authorities have come together in a very relevant White‐paper (Hersey et al., 2022). This is an important development to move from research use only assays to clinically approved assays. Regarding analytical issues the white paper highlights the need for achieving parallelism (Hersey et al., 2022; Lu et al., 2011) and notes situations where there is a lack of parallelism:

- Calibrator and analyte are not recognised in the same way which can be the case in presence of aggregates or diversity of antibodies in the endogenous matrix sample
- Endogenous binding partners interfere
- A matrix effect

Assuming appropriate analytical validation recommendations were made for clinical biomarkers as surrogate endpoints of for patient selection/stratification (Hersey et al., 2022):

- To understand the behaviour of a biomarker in the study population
- To provide analytical and clinical data for proposing what is the best biomarker for providing a clinical endpoint
- To engage early with regulatory agencies
- To carefully and rigorously address the quality control strategy which needs to be sustainable
- To validate the method pre‐analytically (sample collection and processing), analytically (sensitivity, specificity, precision, stability, accuracy, cutoff point) and clinically (agencies requirements, specific context of use, study risk)

The pre‐analytical, analytical and quality control points of this white paper were the subject of the first part of this review. In the following, the clinical aspects are reviewed to address above points regarding clinical studies.

## 11 DISEASES

There has been an impressive increase in diseases and conditions in which Nf have been studied over the past 4 years alone. In this section, the neurological conditions will be reviewed first followed by other medical subspecialties.

### 11.1 Neurological conditions

#### 11.1.1 ALS

This is a rapidly progressive neurodegenerative condition, mostly leading to death within a couple of years. Nf concentrations in the CSF are among the highest observed for any disease (De Schaepdryver et al., 2018; Rossi et al., 2018). Nf stoichiometry changes in ALS (Khalil et al., 2018). The result of adaptive Nf stoichiometry provides a potentially elegant strategy for neurons to save time and energy while maintaining structural integrity (Zucchi et al., 2018). Presymptomatic individuals with known mutations causing ALS to show an increase in blood NfL levels at least 1–3.5 years before clinical onset (Benatar et al., 2019). CSF and blood Nf concentrations permit to identify individuals with rapid and slow disease progression (Brettschneider, Petzold, Suessmuth, et al., 2006; Gille et al., 2018; Lu et al., 2015). Consequently, Nf became a secondary endpoint for two clinical trials in ALS (Miller et al., 2020; Paganoni et al., 2020).

#### 11.1.2 Multiple sclerosis

MS is a chronic inflammatory disease (Thompson et al., 2018). The relevance of axonal pathology in MS was recognised from human post‐mortem samples. This observation heralded much of the pioneering work on Nf biomarker data (Petzold, 2005b). Cumulative, the large number of publications on Nf concentrations indicate that Nf concentrations rise already up to 6 years prior to onset of disease, called the presymptomatic phase. Likewise, CSF and blood NfL and NfH levels indicate the conversion from clinically isolated syndrome (CIS) to RRMS (Brettschneider, Petzold, Junker, & Tumani, 2006; van der Vuurst de Vries et al., 2018). Individuals with the highest Nf concentrations become, undisputedly, more rapidly disabled on a short‐term basis (Brune et al., 2022; Cantó et al., 2019; Petzold, 2005a). They will also experience more severe atrophy on imaging of their brain, spinal cord and retina (Bsteh et al., 2019; Chitnis et al., 2018; Lie et al., 2022; Petzold et al., 2016). The long‐term prognostic value of Nf in MS is more controversial (Manouchehrinia, Stridh, et al., 2020; Sellebjerg et al., 2018). There seems almost no aspect of the known MS pathology left which has not yet been correlated with body fluid levels of Nf isoforms which renders it impossible to include all findings in this review. An elegant recent observation is that elevated blood levels of NfL were related to the presence of paramagnetic rim MRI lesions (Maggi et al., 2021). This observation is consistent with translational evidence for a build up of Nf brain tissue concentrations in the perilesional white matter (Petzold et al., 2008). Release of Nf from ongoing inflammation leading to axonal injury, and proteolytic breakdown of Nf isoforms, in this area is indeed a convenient model to explain high blood Nf levels in individuals with MS in absence of novel contrast‐enhancing lesions. The other explanation is of course retrograde trans‐synaptic axonal degeneration. This has been studied most extensively in the visual system. Credit goes to Jens Kuhle and his team for pioneering a percentile‐based approach guiding the statistical analysis of body fluid Nf levels (Barro et al., 2018; Cantó et al., 2019; Maggi et al., 2021). It is likely that this approach will gain momentum because it permits to eloquently pick out those individuals with pathological Nf levels. Such an understanding is important on many levels including neurotoxicity. Neurotoxicity arising from an intervention can be recognised by Nf (Petzold, Mondria, et al., 2010). Because individuals with MS suffer from an impaired blood–brain barrier (BBB) they may be particularly vulnerable to potentially neurotoxic treatments.

For future study, all of these associations, key challenges are now to clarify how frequently blood Nf levels require measurement and establishment of a clinically meaningful level of change in concentration (Bittner et al., 2021). In addition to the safety, prognostic and monitoring value of Nf in MS there is now phase 3 trial evidence for Nf as a reliable secondary endpoint for immunosuppression (Hauser et al., 2020). The choice of neurofilaments as a successful secondary outcome measure in treatment trials contributed to FDA approval of Ofatunumab for the treatment of individuals suffering from multiple sclerosis on August 20 2020. The use of the NfL Z‐scores also suggests a novel role for plasma NfL levels for remyelination strategies in MS (Abdelhak et al., 2022).

To conclude, there is a relationship between elevated NfL and NfH levels in MS with:

- Disease severity: higher Nf levels indicate more active and progressive disease
- Relapses: higher NfL levels are found for about 3 months after a relapse
- Brain atrophy: higher NfL and NfH levels predict brain atrophy over the following 10–15 years
- Clinical disability scales: correlations with the clinical scales for cognitive and physical functioning which are strongest for involvement of the pyramidal and motor system, presumably due to degeneration of large Nf‐rich axons
- Radiological disease activity: correlations with T2 lesion number and total T2 lesion volume, T1 black holes, contrast enhancing and rim lesions
- Presymptomatic phase of MS: an increase of NfL and NfH levels predict conversion to clinical definite MS
- Conversion of CIS to clinical definite MS
- Overall prognostic: individuals with Nf levels in the higher percentiles have a poorer prognosis than those with Nf levels in the lower percentiles
- Treatment response: successful treatment is followed by a decrease in Nf levels
- Safety laboratory: an elevation of Nf levels during an intervention indicates the potential for neurotoxic side effects. This may be particularly relevant in individuals with NS and a broken BBB.

#### 11.1.3 Neuromyelitis optica

*Neuromyelitis Optica Spectrum Disease* (NMOSD) is an autoimmune channelopathy directed at the water channel Aquaporin 4 (AQP4) located on astrocytes. Damage in NMOSD is complement mediated and occurs during an acute relapse. NfH and NfL concentrations were found to be higher in NMOSD than in MS and to be of prognostic value (Mariotto et al., 2019; Miyazawa et al., 2007). Recently novel biologics became available for the treatment of NMOSD. There is a potential role for Nf for surveillance of individuals on these very expensive drugs. Not only for safety, but also for discovery of sub‐clinical disease activity.

#### 11.1.4 Myelin oligodendrocyte glycoprotein antibody disease

The other novel disease which has entered the clinical arena is *Myelin Oligodendrocyte Glycoprotein Antibody Disease* (MOGAD) (Marignier et al., 2021). Similar to NMOSD damage to the nervous system is relapse related and Nf concentrations are increased (Mariotto et al., 2019).

#### 11.1.5 Stroke

*Acute stroke* affects ≈700,000 people in the US annually (Powers, 2020). Successful implementation of pharmacological thrombolysis and mechanical thrombectomy has substantially improved the outcome. One challenge for clinical trials is that at the time of inclusion one cannot say how much brain tissue has been damaged irreversibly. The pragmatic approach is therefore to make decisions mainly based on the time interval elapsed between onset of stroke and admission. For selecting patients, longer time frames may apply. NfL and NfH concentrations from blood and CSF correlated with survival, short‐ and long‐term clinical outcome, cognitive function and radiological scales (Gendron et al., 2020; Petzold, Worthington, et al., 2011; Sellner et al., 2011; Tiedt et al., 2018; Zhou et al., 2022). Likewise, serum NfL levels were elevated in cerebral autosomal dominant arteriopathy with subcortical infarcts and leukoencephalopathy (CADASIL) (Gravesteijn et al., 2018). There is a role for Nf to optimise planning of patient care pathways and improve on clinical trial efficiency (Gendron et al., 2020).

#### 11.1.6 SAH

In *subarachnoid haemorrhage* (SAH), Nf isoforms (NfL, NfH) are elevated acutely (Garland et al., 2021; Petzold, Keir, et al., 2006; Petzold, Rejdak, et al., 2005). Longitudinal Nf concentrations also permit for early recognition of secondary complications, vasospasm and hydrocephalus, as a change from individual baseline values is observed (Gendron et al., 2020; Petzold, Keir, et al., 2006).

#### 11.1.7 Dementia

One of the biggest challenges for healthcare systems is the increase of dementias in the ageing population, with an estimated prevalence of 45,956,000 patients worldwide (Feigin et al., 2017). The prevalence is expected to rise to 131 million by 2050 and reach 82% in those over 85 years old. CSF biomarkers can precede the onset of dementia by 10–20 years (Bateman et al., 2012). Among the four main clinical categories, Alzheimer's disease (AD), Frontotemporal lobe dementia (FTLD), vascular dementia (SVD), dementia with Lewy Bodies (Pilotto et al., 2021) and minimal cognitive deficit (MCI), Nf concentrations were consistently found to be highest in FTLD (Petzold et al., 2007; Ende et al., 2019).

#### 11.1.8 FTLD

Because early treatment is key, it was recommended for FTLD trials to include NfL as part of a data‐driven selection tool (Ende et al., 2021). The increase in blood NfL levels precedes the onset of FTLD by at least an average of 1.3 years (Gendron et al., 2022). The clinical spectrum of FTLD is heterogeneous and plasma NfL data were highest in FTLD‐ALS compared to individuals with non‐fluent and agrammatic variant primary progressive aphasia (nfvPPA), semantic variant PPA (svPPA). The quantitative, longitudinal, relationship of the serum concentration of NfL and NfH is described in an elegant and carefully conducted multicentre study (Wilke et al., 2021). One question arising from these data relates to the Nf stoichiometry. Presently the reported concentration of NfL in controls (6.6 pg/ml) is about seven times lower than for NfH (47.6 pg/ml), rather than the expected other way round. This observation goes beyond the analytical discussion of the protein standard concentrations used in immuno‐assays. There are four aspects to this finding:

- Is there evidence for endogenous binding of Nf which affects the three isoforms in a different way? This relates, for example to the literature on Nf isoform‐specific autoantibodies in neurological disease?
- Is there a potential role for quantification of NfM which has been absent from most of the studies hitherto reported?
- Can the use of Nf stoichiometry be of use to circumvent statistical issues related to physiological ageing?
- How does modelling of the Nf stoichiometry project on the longitudinal dynamics of Nf isoform concentrations? This with particular reference to the left tail of the polynomial function of the z‐score, twice crossing the zero‐line (Figure 3 A&C in Wilke et al., 2021)

Addressing these and other questions will require a biologically and mathematically logical approach.

#### 11.1.9 AD

In AD PTMs lead to a four to eightfold increase of phosphorylation of NfH and NfM compared to controls (Rudrabhatla et al., 2011). In AD it has been shown that elevated NfL levels are associated with disease progression (Moscoso et al., 2021; Santangelo et al., 2021). In section 7 the relevance of NfL breakdown products, notably in the 10 kDa range (Brureau et al., 2017). Candidate sequences were NfL_92−224_, NfL_324−360_, NfL_530−540_ (Brureau et al., 2017).

- Accelerated cognitive decline
- Higher in presymptomatic individuals with Aβ‐plaques
- Predicts clinical disease onset by 15–20 years
- Related to A*β*‐plaques independent disease progression
- Predicts fast progression
- Improves diagnostic accuracy of AD if added to biomarker panel

#### 11.1.10 DLB

In individuals with Diffuse Lewy Body dementia (DLB) plasma NfL levels are increased if compared to controls (Karantali, Kazis, Chatzikonstantinou, et al., 2021; Pilotto et al., 2021). There is a correlation with cognitive decline.

#### 11.1.11 SVD

Intriguingly in SVD, there is a vasculocentric staining pattern for phosphorylated NfH which was suggested as an alternative passage of the Nf from the brain to the blood (Anad et al., 2022). The increase of Nf concentrations was also more in adult individuals with *Down Syndrome* and related to disease severity including cognitive function (Fortea et al., 2020; Thwaites et al., 2021).

#### 11.1.12 Huntington's disease

(HD) is caused by an expanded CAG repeat in the Huntingtin gene which leads to progressive neurodegeneration. The number of CAG repeats correlated with Nf concentrations (Byrne et al., 2017). Elevated Nf concentrations predicted brain atrophy (Johnson et al., 2018) and disease onset by ≈24 years (Scahill et al., 2020). In two other studies, the prognostic value of blood Nf levels was, however less convincing (Parkin et al., 2021; Wild et al., 2007). This may be related to timing of sampling and longitudinal dynamics of Nf levels (Rodrigues et al., 2020). An increase in serum NfL levels was also associated with increased brain network connectivity in pre‐symptomatic individuals known to harbour HD mutations (McColgan et al., 2022). Finally, Nf has been used as a secondary trial endpoint for a novel antisense oligonucleotide trial in HD (Tabrizi et al., 2019).

#### 11.1.13 Prion disease

In *Prion Disease* (Creutzfeldt Jakob Disease), the process of neurotoxic protein aggregation is so rapid and brain degeneration so widespread that CSF Nf concentrations in Prion disease even eclipse what is observed in ALS (Zerr et al., 2018). Therefore, Nf concentrations are of diagnostic value in the right clinical context (Hermann et al., 2021). Plasma NfL levels were shown to be increased in fatal familial insomnia (Hermann et al., 2022). The highest NfL levels were associated with methionine homozygosity at codon 129 PRNP. Higher NfL levels were of prognostic relevance regarding time to death (Hermann et al., 2022). There is a potential role as a diagnostic biomarker and secondary outcome for treatment trials (Zerr, 2022).

#### 11.1.14 Guillain–Barré syndrome

GBS is a post‐infectious disease affecting the peripheral nervous system (Laman et al., 2022). The disease course of GBS is typically monophasic and can be severe acutely, but most patients make a reasonably good recovery. This is because demyelination in the peripheral nervous system recovers better than in the central nervous system. In a small proportion of individuals with GBS autoantibodies are deposited along the nerve axons (Laman et al., 2022). Overall only a small proportion of individuals with GBS will experience axonal degeneration (Feasby et al., 1986; Petzold, Hinds, et al., 2006). In these patients, elevated CSF Nf concentrations are a poor prognostic sign (Petzold et al., 2002; Petzold, Brettschneider, et al., 2009). It is understood that high CSF Nf concentrations indicate proximal axonal damage at the level of the nerve root (Laman et al., 2022; Petzold, Hinds, et al., 2006). Proximal axonal damage of peripheral motor nerves is less likely to recover than distal axonal damage where axonal sprouting readily occurs (Petzold, Brettschneider, et al., 2009). For this reason, more precise prognostic information is gained from CSF rather than blood Nf levels in acute GBS. For monitoring, however, which requires serial sampling, there is an advantage in quantification of blood Nf levels. This permits early detection of the development of secondary complications (Körtvelyessy et al., 2020; Martín‐Aguilar et al., 2020). Taken together, the Nf data (Körtvelyessy et al., 2020; Martín‐Aguilar et al., 2020; Petzold et al., 2002; Petzold, Brettschneider, et al., 2009; Petzold, Hinds, et al., 2006) contribute to introducing a paradigm shift for the diagnostic workup of diseases seen in a peripheral nerve clinic. It takes 2–3 weeks for neurophysiological tests to diagnose with confidence axonal damage in GBS (see references in Petzold, Brettschneider, et al., 2009), CSF Nf levels provide this information already at onset.

#### 11.1.15 Peripheral neuropathies

Above pioneering work on acute GBS has opened the field of Nf research for other *peripheral neuropathies*. As a rule of thumb, blood Nf levels are higher with acute than with chronic pathology to the peripheral nerve. Blood and CSF Nf levels are helpful with the diagnostic workup, guiding clinical management and trial design. This includes critical illness neuropathy (CIN) (Sandelius et al., 2018), giant axonal neuropathy (GAN) (Bomont et al., 2000), Chronic Inflammatory Demyelinating Polyneuropathy (CIDP) (Kapoor et al., 2022; Karam, 2022; Lieverloo et al., 2019; Mariotto et al., 2018; Petzold, Brettschneider, et al., 2009). There may be a role for plasma Nf isoform levels to interrogate response to treatment (Kapoor et al., 2022). There was a small increase in blood Nf concentrations in individuals with multifocal motor neuropathy (MMN) (Kmezic et al., 2022), paraproteinaemia‐related demyelinating polyneuropathy (Kmezic et al., 2022), Chemotherapy Induced Peripheral Neuropathy (CIPN) (Huehnchen et al., 2022; Karam, 2022; Kim, Choi, et al., 2020) and a range of inherited neuropathies (Kapoor et al., 2019; Millere et al., 2021; Rossor et al., 2016). The highest blood NfL levels were found in individuals with Hereditary transthyretin amyloidosis (ATTRm) and neurological symptoms (65.8 pg/ml) compared to controls (15.5 pg/ml) (Kapoor et al., 2019). These data are very comparable to those obtained from a treatment trial in ATTRm with patisiran (69.4 pg/ml in diseased individuals compared to 16.3 pg/ml in controls) (Ticau et al., 2020). The experience with Charcot–Marie‐Tooth (CMT) illustrates the relevance of increased analytical sensitivity of the immunoassay. The second‐generation ELISA for NfH was found not to be useful (Rossor et al., 2016), while the fourth‐generation SIMOA for NfL was useful (Rossor et al., 2021). There are no data yet on the stoichiometry of NfL:NfM:NfH in these chronic conditions using highly sensitive assays for all Nf isoforms. This may be relevant because it remains to be seen if the effect size for a single Nf isoform from blood samples will be large enough to be considered as a secondary endpoint for treatment trials in CMT, but may be considered for ATTRm (Ticau et al., 2020). This point is not trivial for the ambitious aim for blood Nf levels to join neurophysiology as an equal partner in the diagnostic workup and management of patients with a disease of the peripheral nervous system.

#### 11.1.16 Spinal cord injury

Following spinal cord injury, NfL and NfH levels are increased and appear to respond to treatment (Kuhle et al., 2015). They are also of diagnostic value in the critical initial days after spinal cord injury (Leister et al., 2021). Elevated Nf levels were found helpful for the differential diagnosis between myelitis and spinal cord infarction (Sechi et al., 2021).

#### 11.1.17 Movement disorders

Clinically, the differential diagnosis of *extrapyramidal syndromes* can be challenging. The disease course is slower in *Parkinson's disease* (PD) which progresses over decades if compared to *Progressive Supranuclear Palsy* (PSP) or *Corticobasal Degeneration* (CBD) *Multisystem Atrophy* (MSA) which leads to death in a substantial number of patients in about 3 years. Of the latter, there are again two subtypes, cerebellar (MSA‐C) and pyramidal (MSA‐P). CSF NfL levels are also increased in an extremely rare form of PD in carriers with the E46K‐SNCA mutation (Murueta‐Goyena et al., 2022). The variable penetrance of the PD phenotype in these neurodegenerative disorders renders delineating biomarkers of exceptional value.

Nf help with the differential diagnosis because they are higher in the more severe forms, highest in MSA‐P (Jabbari et al., 2020) and lowest in PD (Lin et al., 2020) with PSP being in between. An early clinical symptom is pure autonomic failure at which time CSF NfL levels are already elevated, preceding conversion to MSA, PD or DLB (Singer et al., 2021). Elevated Nf have prognostic value in rarer movement disorders such as Friedreich Ataxia (Hayer et al., 2020) and Wilson disease (Shribman et al., 2020; Wang, Xu, et al., 2022) which is a curable condition of the copper metabolism. Spinocerebellar Ataxias (SCA) comprise a large number of genetically determined subgroups. Neurofilaments were pathologically elevated in SCA2 and were correlated with disease severity (Yang et al., 2021).

In PD, there are NfL data supported evidence for uncontrolled diabetes mellitus accelerating the progress of neurodegeneration (Uyar et al., 2022). Elevated blood NfL levels were found to precede the onset of PD by at least 5 years (Halloway et al., 2022). The odds ratio for incident PD was 2.54 (95% CI 1.70–3.81) if blood NfL levels were twofold elevated in a model which adjusted for demographic data (Halloway et al., 2022). Blood NfL levels were correlated with disease severity cross‐sectionally and predicted disease progression of cognitive and motor symptoms longitudinally (Lin et al., 2019; Ye et al., 2021). Predictive modelling includes dopamine transporter (DAT) imaging of the putamen (Ye et al., 2021).

#### 11.1.18 Epilepsy

One concern for individuals suffering from *Epilepsy* is neurodegeneration either resulting from seizure activity or as a neurotoxic treatment side‐effect. Indeed, an increase in Nf concentrations has been observed with acute and prolonged seizure activity (Abaroa et al., 2013; Rejdak et al., 2012).

#### 11.1.19 Metastatic brain lesions

Finally, Nf are elevated in structural brain lesions (Hepner et al., 2019; Winther‐Larsen et al., 2020). Elevated NfL levels were related to tumour activity (Hepner et al., 2019). The potential role for monitoring longitudinal Nf blood concentrations to enable early diagnosis of metastatic brain disease (Lin, Lu, et al., 2021; Winther‐Larsen et al., 2020) still needs to be confirmed prospectively.

#### 11.1.20 TBI

*Traumatic Brain Injury* is, with 27.08 million (95% CI 24.30–30.30) new cases alone in 2016, a major cause of death and disability worldwide (James et al., 2019). In *severe* TBI elevated NfL and NfH concentrations give higher odds ratios than clinical scales for predicting morbidity and mortality (Gao et al., 2020a; Kahouadji et al., 2022; Otani et al., 2020; Petzold, Tisdall, et al., 2011; Tisdall & Petzold, 2012). In one study elevation of blood NfL levels persisted for up to 5 years following TBI (Newcombe et al., 2022).

Clinically, it remains challenging to recognise brain damage in *mild* TBI. Neurofilaments acutely elevated in mild TBI (Shahim et al., 2018) and acutely elevated NfL improve on sensitivity with an area under the receiver operating characteristic curve of ≈0.80 and additional prognostic information (Shahim et al., 2018). There is a role for body fluid Nf isoform levels in TBI to:

- Predict mortality
- Predict poor functional outcome
- Early recognition of secondary complications, particularly relevant in a sedated and ventilated patient
- Inform on severity of high impact injury
- Improve sensitivity to detect low impact injury
- Correlate with diffuse axonal injury
- Consequently also correlate with MRI tractography findings

#### 11.1.21 Concussion

Finally, concussions, particularly repeated, pose a challenge to sport and military medicine because of long‐term consequences and disability in exposed individuals (Blennow et al., 2012). A severe, cumulative, consequence is dementia pugilistica or chronic traumatic encephalopathy (CTE). Elevated NfL concentrations are of diagnostic value in the acute and sub‐acute phase. Regarding sport‐related concussion research on Nf included American football, soccer, ice‐hockey, rugby and contact sports (Joseph et al., 2019; Pattinson et al., 2020). The evidence is however not yet conclusive at population level (James et al., 2021) or targeted at top professional football players (Cornali et al., 2022). A word of caution also comes from post‐mortem data which highlight the likelihood of multiple neuropathologies as source for increased body fluid Nf levels (Asken et al., 2022).

### 11.2 Infectious diseases

#### 11.2.1 COVID‐19

During the COVID‐19 pandemic, there have been several reports on elevated Nf concentrations in blood and CSF (Cooper et al., 2020; Edén et al., 2020; Espíndola et al., 2021; Prudencio et al., 2021; Sutter et al., 2021; Virhammar et al., 2021). As a rule of thumb, high body fluid levels of Nf are a prognostically poor sign (Kuhle & Petzold, 2011; Petzold, 2007). Consistent with this notion, longitudinal data on elevated Nf levels predict poor outcome and mortality in COVID‐19 disease (Aamodt et al., 2021; Lorenzo et al., 2021; Prudencio et al., 2021). Regarding research on the neuro‐invasive potential of SARS‐CoV2 there is a role for Nf. This includes research on long COVID‐19 disease.

#### 11.2.2 West Nile Virus

Predating COVID‐19 by about a decade, similar questions surrounded the outbreak of *West Nile Virus* (WNV) (Burki, 2018). This arbovirus infection involved the central and peripheral nervous system. Post‐mortem data showed an inflammatory, perivascular cellular reaction, particularly in the lower brainstem. There was also a relationship between post‐mortem evidence for neuronal death in the brain and spinal cord with CSF Nf concentrations (Petzold, Groves, et al., 2010). This was most notably for motor neurons packed in excess with Nf. There are data to suggest that *flaviviridae*, to which WNV belongs, interact with the cytoskeleton including Nf (Zhang et al., 2019).

#### 11.2.3 Malaria

There is excellent post‐mortem evidence for axonal injury in individuals suffering from cerebral malaria who had been infected with *Plasmodium falciparum* (Medana et al., 2002). The immunohistochemical images show both diffuse and focal axonal injury. This is similar to the two patterns observed in sepsis (Ehler et al., 2017). There was a relationship of the extent of axonal injury with clinical data such as the Glasgow Outcome Score. More recently investigation of this important observation has been translated to investigating plasma NfL levels in a rodent model of malaria (Wai et al., 2022). An association of plasma NfL levels with cerebral oedema was observed, but not related to outcome (Wai et al., 2022). Future human studies building on this work should include clinical signs, such as seizures and coma, which are associated with a 15–20% mortality (Medana et al., 2002). In addition to oedema, such studies should also may also consider the contribution of hypoxia, hypoglycaemia, haemorrhages and inflammation in addition to cerebral oedema (Medana et al., 2002).

#### 11.2.4 Herpes, Lyme, tick‐borne encephalitis, PML & HIV

There are at least five more infectious diseases where elevated Nf concentrations have been associated with neuro‐axonal damage and poorer clinical outcome; *Herpes encephalitis* (HE) (Eckerström et al., 2020; Li et al., 2019; Sellner et al., 2014), *Lyme disease*, *HIV* (Alagaratnam, Francesco, et al., 2021; Gisslén et al., 2016; Mellgren et al., 2007), *tick‐borne encephalitis* (Fortova et al., 2022) and *Progressive multifocal leukoencephalopathy* (PML) (Costa et al., 2019; Toorop et al., 2020). Longitudinal Nf concentrations permit also to monitor development of *PML* in patients that are immunosuppressed (Costa et al., 2019; Loonstra et al., 2019). There is a role for Nf monitoring potential neurodegeneration in the evolving landscape of treatment strategies for HIV which have successfully reduced the likelihood of AIDS‐related dementia (Mellgren et al., 2007). Regarding HIV and AIDS there is a role for body fluid Nf levels to:

- Recognise HIV‐associated dementia, which has become less frequent with effective HIV treatment
- Correlate with cognitive performance
- Recognise HIV‐related polyneuropathy
- Aid in identifying potential neurodegenerative pathology which may occur with HIV treatment strategies
- As a surrogate outcome in HIV treatment trials

### 11.3 Psychiatry

It is crucial to avoid the misdiagnosis of a primary psychiatric disease from a primary neurodegenerative disease. This is one important diagnostic role for Nf (Ducharme et al., 2020). Higher Nf concentrations are observed with neurodegeneration. It is, therefore, interesting to note that there is a small increase in Nf concentrations in *Depression* (Bavato et al., 2021; Chen, Liu, et al., 2021; Zhao et al., 2020), *Schizophrenia* (Bavato et al., 2021; Rodrigues‐Amorim et al., 2020) and with *Psychosis* (Katisko et al., 2019). Future research will need to investigate how much of this is due to neuro‐axonal loss or potential side effects of treatments. Normal Nf concentrations following electro‐convulsive therapy for severe depression have been helpful for monitoring safety of this treatment option (Besse et al., 2020).

### 11.4 Cardiology

#### 11.4.1 Cardiac arrest

Cardiac arrest causes neurological disability among survivors in about a third. Blood NfH and NfL concentrations are of prognostic value in predicting survival and anticipating neurological impairment (Hunziker et al., 2021; Moseby‐Knappe et al., 2019; Rundgren et al., 2012) and cognitive impairment (Nordström et al., 2022). Recognised to be more sensitive compared to other blood biomarkers, Nf have been included as secondary outcome measure in two trials aiming for an increased mean arterial pressure (Perkins et al., 2021). These studies also highlighted that a pre‐existing neurological comorbidity is a relevant bias. Contemporary recommendations are to include Nf as a blood biomarker for brain injury in trials alongside the clinical examination, electrophysiology and brain imaging (Perkins et al., 2021).

#### 11.4.2 Arrhythmia and surgery

Extending on cardiac arrest there is a prognostic role for Nf isoforms (NfL and NfH) in *Atrial fibrillation* (Polymeris et al., 2020) and in *cardiac surgery* (DiMeglio et al., 2019; Hunziker et al., 2021; Rundgren et al., 2012). Elevated Nf isoforms in the CSF or blood are a poor prognostic sign in cardiac arrest. Monitoring Nf concentrations aided in assessing safety parameters for cardiopulmonary bypass (CPB) surgery (Wiberg et al., 2020).

### 11.5 Critical care

*Sepsis* and *septic shock* are serious problems in critical care because of the high level of morbidity and mortality. Recognition of septic encephalopathy is difficult in the sedated, intubated and ventilated patient. Because of this and the frequently unstable haemodynamic situation, transport of patients to a scanner for brain imaging is logistically extremely challenging and frequently not possible because of safety concerns. There is translational research spanning from experimental models of sepsis, over human post‐mortem examination to Nf analysis which strongly suggests Nf to be a specific biomarker for the two patterns of brain damage occurring in sepsis (Ehler et al., 2017). First, there are small embolic insults which can also be observed on brain imaging (Ehler et al., 2019). Second, there is diffuse axonal damage which is not captured by imaging but a likely source of elevated blood Nf concentrations arising from proteolytic breakdown products (Ehler et al., 2019; Orhun et al., 2021). The findings of body fluid Nf isoform levels relating to severity of brain function impairment seen in septic encephalopathy expands to a milder form, termed *delirium* (Fong et al., 2020; Halaas et al., 2018; Inoue et al., 2017; Mietani et al., 2019).

### 11.6 Gastroenterology

There is an important link between impaired liver function and neurological symptoms caused by metabolic encephalopathy. Serum NfL levels are a sensitive biomarker for minimal hepatic encephalopathy (Labenz et al., 2021). The median levels were about threefold above the normal control values of 24.3 pg/ml. The association of diarrhoea with chronic systemic symptoms involving other medical specialities is a ‘red flag’ for *amyloidosis* (Cabot et al., 1985). Pathological blood NfL concentrations in amyloidosis are of diagnostic value for involvement of the nervous system (Louwsma et al., 2020; Ticau et al., 2020). In oesophageal cancer surgery, an elevation of serum NfH levels was predictive of post‐surgery delirium (Mietani et al., 2022). Elevated plasma NfL levels have been reported anorexia nervosa (Nilsson et al., 2019; Wentz et al., 2020).

### 11.7 Ophthalmology

In acute *optic neuritis* (ON) higher Nf blood concentrations were related to poorer visual function and treatment response (Petzold et al., 2004). In the Optic Neuritis Treatment Trial, there was a similar, albeit weaker association for blood NfH levels collected in the later stage of ON (Pasol et al., 2010). In ON, the release of Nf from axons originates from axons along the entire visual pathways through the human brain. The highest degree of optic disc pallor developed in those individuals with the highest blood Nf concentrations (Petzold, Boer, et al., 2010). This clinical observation has since been confirmed by state‐of‐the‐art optical coherence tomography studies (Bsteh et al., 2019; Mariotto et al., 2019; Seitz et al., 2021; Tavazzi et al., 2020). There is experimental evidence for early up‐regulation of retinal tissue Nf levels after axonal damage in ON (Weissert et al., 2022). Next, anterograde axonal transport of Nf isoforms, and their proteolytic fragments, from the retina to the optic nerve contributes to the sustained high blood Nf isoforms concentrations (Mariotto et al., 2019; Petzold et al., 2004).

The other body fluid compartments adjacent to the retinal axons are the vitreous body and anterior chamber fluid. Elevated Nf concentrations can also be observed in these compartments (Petzold, Junemann, et al., 2009; Subramanian et al., 2020; Woltsche et al., 2022). Observations made in *Retinal Detachment* (RD) (Petzold, Junemann, et al., 2009), *Age‐related Macular degeneration* (AMD) (Nielsen et al., 2019) and glaucoma (Woltsche et al., 2022) are of potential interest for investigation of longitudinal Nf concentrations from these compartments in other ophthalmological conditions. Big public health issues such as glaucoma and diabetic retinopathy are obvious candidates.

### 11.8 Obstetrics and gynaecology

The advance in surgical techniques permitted for prenatal repair of myelomeningocele (Adzick et al., 2011). The approach stimulated an extensive debate and Nf may contribute to addressing open questions. Experimentally amniotic fluid Nf concentrations were related to the extent of prenatal spinal cord injury in an animal model with naturally occurring *Menignomyelocele* (Petzold, Stiefel, & Copp, 2005). Longitudinal blood Nf concentrations provide a much more convenient way for potential screening of candidates eligible for surgery and monitoring of progress. Blood Nf concentrations were already successfully quantified in *pre‐eclampsia* (Evers et al., 2018). The data on Nf concentrations in normal pregnancies in this study, however, imply that there will have to be separate normal Nf values for pregnant women.

### 11.9 Oncology

Neurodegeneration is a recognised feature of paraneoplastic disease. It cannot be overemphasised how important the newly described association of increased blood Nf levels in this context might be (Mizenko et al., 2021; Zoccarato et al., 2021). This is because a challenge for neuro‐immunology remains the limited number of validated paraneoplastic antibodies, but frequently a high clinical suspicion of an association remains in individuals who do not harbour any of the known paraneoplastic antibodies. Future studies are required to test for a potential role of CSF and blood Nf levels as an additional paraclinical test for paraneoplastic disorders causing neurodegeneration of the central and or peripheral nervous system.

The role of Nf levels in oncology, more generally, is likely to be limited to structural lesions (Hepner et al., 2019; Winther‐Larsen et al., 2020). For example a recent study on chemotherapy in breast cancer did not find that there was an increase in blood Nf levels (Argyriou et al., 2021). Earlier work suggested that a requirement for chemotherapy to induce neurodegeneration is an impaired blood–brain barrier (Petzold, Mondria, et al., 2010).

### 11.10 Paediatrics

The role of Nf in paediatric disease recapitulates largely what has been observed for adults. The main laboratory difference is the need for age‐adjusted Nf normal values. The paediatric disease spectrum broadens to include many genetic disorders.

Specifically, Nf blood levels have been studied in *Mitochondrial disorders* (Varhaug et al., 2021), *CLN3* (Do et al., 2020), *Autism Spectrum Disorder* (He et al., 2020), *Spinal muscular atrophy* (SMA) (Darras et al., 2019; Faravelli et al., 2020; Kong et al., 2021; Nitz et al., 2021), *Congenital Heart Disease* (CHD) (Lee et al., 2018), *Beta‐propeller protein‐associated neurodegeneration* (BPAN) (Takano et al., 2017), *Gangliosidosis* (Welford et al., 2022), *Febrile seizures* (Evers et al., 2020), *myotonic dystrophy* (Nicoletti et al., 2022), *Ceroid lipofuscinosis type 2 (CLN2)* (Ru et al., 2019) and *Langerhans cell histiocytosis* (Sveijer et al., 2022). Plasma NfL levels are over 10‐fold elevated in the gangliosidosis GM1 and GM2 if compared to controls (Welford et al., 2022). Plasma NfL levels were highest in the first decade of life, probably owing to a more severe phenotype at this age. Samples taken in the thirties were only marginally elevated compared to healthy controls.

With novel treatment options (e.g. SMA, CLN2) and questions on long‐term clinical outcomes, there is an important role for Nf to contribute with long‐term monitoring. There are direct implications for the use of NfL and NfH levels for the monitoring of diseases progression in children with SMA who receive nusinersen (Darras et al., 2019; Ru et al., 2019). The long‐term decrease of CSF NfH levels is small, and was not significant for CSF NfL or either Nf isoform in the serum (Wel et al., 2022).

### 11.11 Pulmonology

Because of their high metabolic demand neurons are vulnerable to hypoxia. Obstructive sleep apnoea syndrome (OSAP) is a known risk factor for ischaemic damage to the retina and brain. Is is therefore interesting to learn that in OSAP averaged serum NfL levels were at group level minimally higher if compared to controls (Arslan et al., 2021).

### 11.12 Rheumatology

Recognition of rheumatological treatment‐related complications (e.g. PML) using Nf was already discussed. In addition, Nf concentrations were found to be elevated and to be of prognostic relevance in *Sjogren's syndrome and Lupus* (Tjensvoll et al., 2020; Zervides et al., 2022) and *Vasculitis* (Bischof et al., 2017; Pawlitzki et al., 2019).

### 11.13 Recreational drug use

The use of ‘Ecstasy’ or 3,4‐Methylenedioxymethamphetamine (MDMA) has been linked to neurotoxicity clinically and experimentally with elevated NfL levels providing an indirect means for measurements (Bavato, Stamatakos, et al., 2022). Likewise, there is an increase in blood NfL levels with ketamine dependence (Liu et al., 2021) and in chronic cocaine use (Bavato, Kexel, et al., 2022). Reduction of use in cocaine was monitored by cocaine hair concentrations and was correlated over a 4‐month period with reduction of blood NfL concentrations (Bavato, Kexel, et al., 2022).

### 11.14 Space flight

There is experimental evidence for space flight inducing a decrease in Nf immunoreactivity in mechanoreceptors (Proshchina et al., 2022). This observation has been linked to the hypogravitatational motor syndrome (HMS), a subform of the space adaptation syndrome (SAS). Neuronal and axonal changes to the staining pattern for NfH were observed already within 12 days after space flight (Proshchina et al., 2022). Interestingly, space flight also changes the gene for NfL in plants (*Arabidopsis*) (Shymanovich et al., 2022). There are data indicating a minute increase in blood NfL levels in five cosmonauts from 11.4 pg/ml preflight to 15.2 pg/ml on return (*p* = 0.04) and a further increase over the following week to 17.2 pg/ml (*p* = 0.04) (Eulenburg et al., 2021). The group average remains within the normal range and it remains to be seen if the small variation over time of 3.8–5.8 pg/ml are over and above to what can be expected from physiological variation of between days 6.6 pg/ml (range 2.6–17.5) or variation within the same day averaging at 7 pg/ml (Hviid et al., 2021). Therefore, future, prospective, longitudinal studies are required to answer if these observations are caused by brain injury (Eulenburg et al., 2021), changes to Nf gene expression (Shymanovich et al., 2022), or release of Nf proteins from the periphery (Proshchina et al., 2022).

### 11.15 Other factors affecting Nf values

The interpretation of Nf concentrations in the elderly can be complex because, in addition to age, comorbidities such as impaired renal function, body mass index and presence of diabetes mellitus might be relevant (Akamine et al., 2020; Bakovic et al., 2018; Fitzgerald et al., 2022; Manouchehrinia, Piehl, et al., 2020; Uyar et al., 2022). Others did not find such an association with renal function in individuals with secondary progressive MS (Williams et al., 2021). Haemodialysis increased plasma Nf levels (Chen et al., 2022). Elevated Nf levels are present too little (Wentz et al., 2020) and too much nourishment (Beydoun et al., 2021; Manouchehrinia, Piehl, et al., 2020). The longitudinal concentration of Nf normalised with successful regain of weight (Doose et al., 2021). Related to at least diabetes mellitus, an association was found between elevated serum NfL levels that were predictive of all‐cause mortality in women (Beydoun et al., 2022).

## 12 META‐ANALYSES

There have been 33 meta‐analyses on the wealth of data on Nf as a biomarker for neurodegeneration over the past 15 years (Agah et al., 2018; Alagaratnam, Widekind, et al., 2021; Angelopoulou et al., 2021; Arneth & Kraus, 2022; Cai & Huang, 2018; Choong et al., 2019; Cong et al., 2021; Forgrave et al., 2019; Gao et al., 2020b; Ge et al., 2018; Hoiland et al., 2022; Hu et al., 2017; Jin et al., 2019; Karantali, Kazis, Chatzikonstantinou, et al., 2021; Karantali, Kazis, McKenna, et al., 2021; Katayama et al., 2020; Koychev et al., 2021; Li et al., 2016; Liu et al., 2022; Liu, Chen, et al., 2020; Martin et al., 2019; Mondello et al., 2021; Peng et al., 2022; Petzold et al., 2007; Rübsamen et al., 2022; Sako et al., 2015; Sferruzza et al., 2021; Wang et al., 2019; Wang et al., 2020a; Wang et al., 2020b; Xu et al., 2016; Zhang et al., 2022; Zhao et al., 2019; Zhou et al., 2021).

### 12.1 Dementia

#### 12.1.1 AD

Regarding AD (*n* = 3138) CSF NfL were of higher concentration than in healthy control subjects (n‐1230) with a good ratio of means (ROM) of 2.12 and narrow 95% CI (1.85–2.42) of the 29 studies included (Forgrave et al., 2019). The ROM was smaller (1.18; 95% CI 1.11–1.25) for the comparison of individuals with AD (*n* = 442) to those with MCI (*n* = 545) in data from eight studies (Forgrave et al., 2019). Unfortunately, CSF NfL levels were higher in a group of AD disease mimics (*n* = 1647) if compared to AD (*n* = 2404) with a ROM of 0.87 (95% CI 0.70–1.08) (Forgrave et al., 2019). The last finding highlights a problem with the interpretation of CSF NfL levels as a diagnostic biomarker for dementia.

Similar was observed for blood NfL levels, higher in AD (*n* = 471) if compared to cognitively unimpaired controls (*n* = 518) with a ROM of 2.61 (95% CI 1.54–4.44) (Forgrave et al., 2019). For the comparison of AD (*n* = 313) with with MCI (*n* = 381) the ROM (1.30) was not significant with the 95% CI crossing the value ‘one’ (0.86–1.95) (Forgrave et al., 2019).

#### 12.1.2 FTLD

The signal for CSF NfL levels was found to be highest for patients with FTLD (*n* = 56) compared to controls (*n* = 98, 1.38; 0.99–1.77), with a very small effect size for CSF NfL only to separate FTLD (*n* = 58) from AD (*n* = 99, 0.62; 95% CI 0.26–0.97) (Petzold et al., 2007). For CSF NfH the effects were smaller than for CSF NfL for FTLD (*n* = 59) compared to controls (*n* = 108, 0.74; 95% CI 0.40–1.08) and not significant for FTLD compared to AD (Petzold et al., 2007). The main findings were confirmed subsequently on larger patient numbers in a meta‐analysis focused on NfL only (Karantali, Kazis, Chatzikonstantinou, et al., 2021). The effect size for CSF NfL in FTLD (*n* = 942) compared to controls (*n* = 696) was very high 2109.10 (95% CI 1527.69–2690.50). More concerning, 13 studies on FTLD were not included in one meta‐analysis (Figure 4 in (Karantali, Kazis, Chatzikonstantinou, et al., 2021)) 2 years after these data were compiled (Figure 3a in (Forgrave et al., 2019)) and four studies were not included in a meta‐analysis 14 years earlier (Figure 3b in (Petzold et al., 2007)). Therefore, the 2019 meta‐analysis should presently be regarded as the most authoritative one with an effect size of 3.41 (95% 2.96–3.93) comparing FTLD (*n* = 1827) with controls (*n* = 1113) (Forgrave et al., 2019).

#### 12.1.3 MCI

One meta‐analysis specifically addressed the issue of MCI collected from seven studies (Zhang et al., 2022). Published 3 years after a meta‐analysis based on blood NfL levels in CIS (*n* = 381) (Forgrave et al., 2019), the 2022 meta‐analysis^1^ pooled CSF NfL and blood NfL data from subjects with MCI (*n* = 676) and controls (*n* = 504) (Zhang et al., 2022). The effect size was 0.36 (95% CI 0.04–0.68) (Zhang et al., 2022). Subsequent subgroup analysis did confirm a significant effect for CSF NfL levels 0.61 (95% CI 0.21–1.01), but did not find blood NfL levels to be of diagnostic value in MCI (Zhang et al., 2022).

### 12.2 Movement disorders

#### 12.2.1 PD

Four meta‐analyses included data on PD (Angelopoulou et al., 2021; Ge et al., 2018; Hu et al., 2017; Sako et al., 2015). Another two meta‐analyses are planned (Wang et al., 2020a; Wang et al., 2020b). A potentially diagnostically interesting increase of CSF NfL in individuals with PD (*n* = 1035) from those with atypical parkinsonian syndromes (*n* = 930) was found (Angelopoulou et al., 2021). The difference of 1.26 standard deviations is of interest for studies operating on a group level and expands on an earlier meta‐analysis on four studies (1.60; 95% CI 1.22–1.98) (Sako et al., 2015). The differential use of a fixed‐effect model ($I^{2}<$ 50%) versus random‐effect model ($I^{2}<$ 50%) was announced as depending on the degree of statistical heterogeneity $I^{2}$ in one protocol for blood NfL (Wang et al., 2020b) which reads similar to the protocol published for CSF NfL (Wang et al., 2020a).^2^

One study (Ge et al., 2018) made use of a hierarchical summary receiver‐operating characteristic model which helps to visualise sensitivity and specificity levels of a test (Rutter & Gatsonis, 2001). This meta‐analysis included 8 studies and found a diagnostic sensitivity for NfL to separate individuals with PD from atypical parkinsonian syndromes of 0.82 (95% CI 0.64–0.91). The corresponding diagnostic specificity was 0.85 (95% CI 0.79–0.89) (Ge et al., 2018).

The comparison of PD with MSA subject to the remaining meta‐analyses is summarised in the next paragraph (Hu et al., 2017).

#### 12.2.2 MSA

MSA (Cong et al., 2021; Hu et al., 2017). There was a highly significant and robust increase of CSF NfL (2500.99, 95% CI 2212.28–2789.70) in MSA (*n* = 142) if compared to controls (*n* = 215) from five studies between 2015 and 2018 (Cong et al., 2021). Likewise, CSF NfL was significantly higher (805.23, 95% CI 672.37–938.10) in MSA (*n* = 77) if compared to PD (*n* = 86) using a fixed model on the mean difference (Cong et al., 2021). If one uses instead a random model on the mean differences (Hu et al., 2017), the findings are largely confirmed, but the reported effect size is smaller. CSF NfL levels were higher in MSA (*n* = 212) if compared to PD (*n* = 373) at 1.56 (1.12–2.00) based on nine studies (Hu et al., 2017).

#### 12.2.3 CJD

Unique among the meta‐analysis discussed here is the network meta‐analysis approach used for CJD (Rübsamen et al., 2022). The method is elegant as it permits ready comparison of sensitivity and specificity values for a range of biomarkers across studies, but does not require that all studies had to include all of the biomarkers. Inclusion criteria were strict, drilling down to the analytical techniques used, cutoff levels reported and details of the patient cohorts. The point was made that while CSF NfL had the highest diagnostic sensitivity (0.99) but not specificity for different diagnostic categories of CJD (Rübsamen et al., 2022). The use of plasma and CSF NfL in CJD has been validated for the early disease course (Schmitz et al., 2022).

#### 12.2.4 Genetic ataxia

Out of 11 studies on blood NfL levels in genetic ataxias, eight used fourth‐generation (SIMOA) immuno‐assays and were included to a meta‐analysis (Peng et al., 2022). The highest effect size was found for ataxia telangiectasia (*n* = 38) if compared to healthy controls (*n* = 20, 1.70; 95% CI 1.41–2.00) (Peng et al., 2022). This was followed by individuals with SCA3 (*n* = 464) where blood NfL levels were higher (1.23; 95% CI 1.18–1.29) if compared to healthy controls (*n* = 828) (Peng et al., 2022). The effect sizes were smaller for SCA1 (*n* = 23) compared to healthy controls (*n* = 25, 1.00; 95% CI 0.39–1.60); Friedreich ataxia (*n* = 217) compared to healthy controls (*n* = 62, 0.62; 95% CI 0.21–1.03) and SCA2 (*n* = 16) compared to healthy controls (*n* = 22, 0.59; 95% CI 0.27–0.91) (Peng et al., 2022). For SCA3, higher blood NfL levels were found in young mutation carriers (Garcia‐Moreno et al., 2022).

### 12.3 ALS

Five meta‐analyses included data on ALS (Forgrave et al., 2019; Li et al., 2016; Sferruzza et al., 2021; Xu et al., 2016; Zhou et al., 2021). Similar to PD (Wang et al., 2020a, 2020b) there is an ongoing, protocolised meta‐analysis (Agah et al., 2018). The most complete meta‐analysis on disease (*n* = 16 studies) comparison demonstrated higher blood NfL levels in individuals with ALS (*n* = 930) if compared to cognitively unimpaired controls (*n* = 593) with an effect size of 9.64 (95% CI 6.65–13.99) (Forgrave et al., 2019). The effect size was smaller (3.35; 95% CI 2.19–5.12) for blood NfL levels in ALS (*n* = 1239) if compared to disease mimics (*n* = 806, 11 studies) (Forgrave et al., 2019). The first meta‐analysis was based on sensitivity and specificity levels (both 0.8) including 11 studies (Li et al., 2016). Detailed subgroup analysis were performed in a meta‐analysis including 15 studies for NfL (4 studies) and NfH (11 studies) in (Xu et al., 2016). The effect size for blood NfL levels was 1.45 (95% CI 1.24–1.66) comparing individuals with ALS with controls. This effect size is smaller than what was found in the later, larger dataset to which more of the fourth generation immuno‐assays contributed (Forgrave et al., 2019). A subsequent meta‐analysis^3^ included only 12 studies (Sferruzza et al., 2021), which is less than what was included earlier (Forgrave et al., 2019; Xu et al., 2016). This meta‐analysis also reports lower effect sizes for blood NfL (1.59; 95% CI 1.25–1.92) levels in ALS (*n* = 1074) compared to healthy controls (*n* = 707) (Sferruzza et al., 2021) with respect to the earlier performed meta‐analysis on a larger dataset with an about six times larger effect size of 9.64 (Forgrave et al., 2019). The effect size is lower 0.72 (95% CI −0.39 to 1.83) for the comparison of ALS (*n* = 539) with other neurological disease controls (*n* = 216); or of ALS (*n* = 655) with ALS mimics (*n* = 358) at 0.95 (95% CI 0.42–1.48) (Sferruzza et al., 2021).

Finally, one meta‐analysis calculated the risk for disease progression and earlier death in ALS (Zhou et al., 2021). This meta‐analysis included 14 studies and 1619 individuals with ALS. Nf levels were grouped according to quartiles. There was an increased risk of faster disease progression and earlier death with higher NfL and pNfH levels. The effect size for faster disease progression was 0.59 (95% CI 0.49–0.69) for NfL and 0.56 (95% CI 0.25–0.88) for pNfH (Zhou et al., 2021).

### 12.4 MS

There are two meta‐analyses on NfL data in MS (Cai & Huang, 2018; Martin et al., 2019). A 0.88 (95% CI 0.05–1.26) increase in CSF NfL was found in MS (*n* = 469) if compared to controls (*n* = 326) in an analysis including 10 studies. Data from these studies were based on a range of different assays spanning 21 years (1996–2021) of research (Cai & Huang, 2018). In contrast, data on blood NfL concentrations were based on the same test used by all five studies included resulting in a lesser degree of heterogeneity with a 0.47 (0.24–0.71) increase in MS (*n* = 1196) compared to controls (*n* = 660). These authors did not find matching groups for to be a major factor either in the random‐effect model or meta‐regression (Cai & Huang, 2018). Gender however was, and so was sample size, but paradoxically inverse to the expected (Cai & Huang, 2018). The second meta‐analysis^4^ found a roughly comparable effect size for CSF NfL levels (0.61; 95% CI 0.48–0.73) in relapsing–remitting MS (*n* = 746) if compared to a mix of healthy and diseased controls (*n* = 435) from data based on 13 studies (Martin et al., 2019). These authors also confirmed by meta‐analysis the observation that CSF NfL levels are higher during a relapse in MS if compared to patients who remain in remission (Martin et al., 2019). The effect size is increased (0.96; 95% CI 0.72–1.20) if only patients with a progressive disease course (*n* = 158), secondary or primary, are compared to controls (*n* = 172) (Martin et al., 2019). The treatment effect of natalizumab on NfL levels was meta‐analysed from seven trials (Liu et al., 2022). NfL levels were lower in natalizumab treated (*n* = 947) compared to pre‐treatment levels (*n* = 959). The effect size was 0.73 (Liu et al., 2022).

### 12.5 Brain injury, TBI and concussion

Brain injury and TBI were subject to two meta‐analyses (Gao et al., 2020b; Mondello et al., 2021). The outcomes of these two meta‐analyses are diametrically opposed. The provocative conclusion of the 2021 meta‐analysis was that ‘There is insufficient evidence to support the clinical validity of initial circulating c‐Tau or neurofilament protein concentrations for the management of patients with mTBI’. (Mondello et al., 2021). In contrast, the 2020 meta‐analysis^5^ found NfL levels to be increased (2.48; 95% CI 1.52–3.43) in TBI (*n* = 569) if compared to controls (*n* = 549) based on data from nine studies (Gao et al., 2020b). A subgroup analysis revealed that ethnicity was relevant. Further subgroup analyses showed that the effect sizes were larger in earlier samples after the event 2.89 (48 h), 2.98 (6–10 days) and 0.28 (1–3 months). Interestingly using a second‐generation immuno‐assay gave an overall larger effect size of 4.06 (95% CI 1.22–6.91) if compared to the fourth‐generation immuno‐assay (1.35; 95% CI 0.79–1.92), but this seems to be driven by two out of six studies, with an overall more homogeneous data distribution of the fourth‐generation immuno‐assay (Figure 3b in (Gao et al., 2020b)). Not surprisingly the effect size was larger for CSF NfL levels (4.03; 95% CI 0.45–7.61) if compared to either serum (2.35; 95% CI 0.45–4.26) or plasma (1.06; 95% CI 0.50–1.61) (Gao et al., 2020b).

### 12.6 Stroke

One meta‐analysis included five studies on blood NfL levels in stroke using third‐ and fourth‐generation immuno‐assays (Liu, Chen, et al., 2020). The meta‐analysis was focused on poor functional outcome. The odds ratio for increased NfL levels predicting poor functional outcome in individuals with stroke (*n* = 1346) was 1.71 (95% CI 1.17–2.49).

### 12.7 Cardiac arrest

One meta‐analysis tested the prognostic value of a range of biomarkers in cardiac arrest (Hoiland et al., 2022). The summary receiver operating curve characteristic analyses (Figure 2a–f in (Hoiland et al., 2022)) showed an AUC of 0.92 for blood NfL levels (*n* = 935) for predicting unfavourable outcome. This was a better AUC compared to NSE (0.84, *n* = 4898), S100B (0.85, *n* = 1724), GFAP (0.77, *n* = 911), tau (0.89, *n* = 792) or Ubiquitin carboxyl hydrolase L1 (0.88, *n* = 702). These data are consistent with an AUC of 0.96 for NfL in a smaller study (Meyer et al., 2022). As demonstrated before for TBI (Gao et al., 2020b), timing of sampling was relevant with blood NfL levels continuing to rise from 24 to 72 h after the event (Figure 3d in (Hoiland et al., 2022)).

### 12.8 Relationship of CSF and blood NfL levels

A pooled correlation coefficient meta‐analysis showed that there is a strong association between CSF NfL and blood NfL concentrations with *R* = 0.723 (95% CI 0.540–0.840) based on data from 36 studies (*n* = 3961 samples) (Alagaratnam, Widekind, et al., 2021). There was a significant heterogeneity between the studies included.

### 12.9 Meta‐analysis summary

Taken together most meta‐analyses were focused on NfL in the CSF and blood. They consistently confirm that higher NfL values are found in the experimental (diseased) group. Factors found to influence these data were age, disease duration and disease severity. For future meta‐analyses, it is advised to strive for completeness of inclusion of studies (Forgrave et al., 2019; Karantali, Kazis, Chatzikonstantinou, et al., 2021; Sferruzza et al., 2021). It was unexpected to find differences as large as a factor of six for the effect sizes reported by different meta‐analyses on the same disease published only 2 years apart (Forgrave et al., 2019; Sferruzza et al., 2021). There is an unfortunate lack of later meta‐analyses referring to earlier work on the same disease. There is a need for justification of using a fixed‐effect versus random‐effect model (Wang et al., 2020b) and a ROM‐based approach is preferred (Forgrave et al., 2019). The ROM approach overcomes some of the statistical limitations of other meta‐analytical approaches (Forgrave et al., 2019). The ROM can be understood as a x‐fold change of Nf concentrations, with values above one *(this is in difference to the effect size for which this value would be zero)* indicating higher values in the group of interest. This approach permits to correct for errors identified by the authors, such as: ‘several studies were observed to misreport units for NfL […]’ (Forgrave et al., 2019). This ROM‐based meta‐analysis also carefully reviewed the analytical methods used for quantification of Nf, importantly including the pair of Nf‐antibodies used for capture and detection. There are good reasons for reporting these antibodies and inclusion to a nomenclature was proposed (Petzold et al., 2003), which was adopted for a period of time (Gaiottino et al., 2013; Kuhle et al., 2010; Petzold & Shaw, 2007). Worryingly, 13/65 (20%) studies included did not report which Nf antibodies were used (Forgrave et al., 2019) which makes it very difficult to compare results other than by the ROM approach.

There will be a need for future meta‐analyses to compare the performance of different biomarkers (Rübsamen et al., 2022). This is because health care sustainability requires an economic use of resources and tests which ultimately will depend on the clinical utility expressed as sensitivity and specificity. A network‐based meta‐analysis approach seems promising for head‐to‐head comparisons which include different Nf isoforms, Nf phosphoforms and Nf proteolytic breakdown products (Rübsamen et al., 2022). In addition, the meta‐analyses demonstrated that there are now more data on blood than on CSF Nf concentrations. This is a clear win attributed to the excellent detection limit of fourth‐generation immuno‐assays. Analyses of longitudinal data become possible. The meta‐analyses also remind us that neither CSF nor blood Nf levels are diagnostic for one disease. Accepting that Nf is not a disease‐specific biomarker, it remains relevant for prognosis (Zhou et al., 2021). Therefore, a pertinent question put to future meta‐analyses is: ‘what is the prognostic value of elevated Nf levels’? and expanding on this ‘what is the prognostic value of sustained or rising NfL levels over time’? For conducting such future meta‐analyses it will be relevant to have well‐defined clinical outcome measures to interrogate prognosis.

The large number of studies on body fluid biomarkers has made it almost impossible to cover all relevant data in a future single review. What will remain possible are systematic reviews on select disease groups or reviews drawing on meta‐analyses. The data reviewed here imply that there is a need for developing reporting guidelines for robust meta‐analyses which take into account relevant pre‐analytical, analytical and clinical factors. An alphabetical overview of the 87 conditions included to this review is presented in Table 3.

**TABLE 3:** Diseases and conditions in which Nf isoforms have been quantified in the human brain interstitial fluid, CSF, plasma, serum, amniotic fluid, anterior chamber fluid and vitreous. This review included 87 conditions and their clinical subtypes. This list is expected to expand

AD	DLB	MS
AF	Epilepsy	MSA
AIDS	Oesophageal cancer surgery	Myotonic dystrophy
ALS	Febrile seizures	NMOSD
AMD	Friedreich Ataxia	ON
Amyloidosis	FTLD	OSAP
Anorexia nervosa	FTLD‐ALS	Paraneoplastic disease
ATTRm	GAN	PD
Autism Spectrum Disorder	Gangliosidosis	PML
BPAN	GBS	PPA
Cardiac arrest	Glaucoma	Pre‐eclampsia
Cardiac surgery	HD	PSP
CBD	Hepatic encephalopathy	Psychosis
CHD	Herpes encephalitis	RD
Chemotoxicity	High BMI	SAH
CIDP	HIV	SAS
CIN	HMS	SCA
CIPN	Ketamine dependence	Schizophrenia
CJD	Kidney function impairment	Sepsis
CLN2	Langerhans cell histiocytosis	Sjogren's syndrome
CLN3	Lupus	SMA
CMT	Lyme	Spinal cord injury
Cocaine use	Malaria	Stroke
Concussion	MCI	SVD
COVID‐19	MDMA use	TBI
CTE	Menignomyelocele	Tick‐borne encephalitis
Dementia	Metastatic brain lesions	Vasculitis
Depression	MMN	Wilson disease
Diabetes mellitus	MOGAD	WNV

## 13 STATISTICS AND TRIALS

There is a need to further develop the statistical approach. A likely influential paper incorporated for example NfL, age and the body mass index (BMI) values into z‐scores. One advantage of this approach is that bold comparisons can be made. The NfL Z‐score data strongly suggest a differential effect of treatment options on blood NfL levels in MS (Benkert et al., 2022). There is no doubt that Nf levels are useful on a group level. The question now is if they will become useful on an individual subject level?

For clinical trial design, it will be relevant to note that all neuroprotective treatment trials in MS which had failed on these primary outcome measures, now retrospectively showed significant effects regarding Nf levels (Preziosa et al., 2020).

Power calculations for trial design will need to include data on the inter‐ and intra‐assay CV, physiological variation of Nf isoforms. A large analytical error, caused for example by a large intra‐assay CV will mask small changes in Nf levels over time. While Z‐scores can correct for demographic data and comorbidities, they remain vulnerable to analytical errors.

## 14 OUTLOOK

To continue its trajectory, the ‘dazzling rise of Nf […]’ (Bomont, 2021) needs further acceleration. On top of the increasing quantity of clinical studies focused on the subject of this review, there are fundamental questions to basic sciences.

1. There is half a century of research on the presence of autoantibodies directed at Nf (Petzold, 2005b). Several PTMs of Nf isoforms reviewed act as a trigger for autoimmunity. Until now, the role of such autoantibodies remains unclear. Clinically, it will be important to find out if they are of pathological relevance or only an epiphenomenon? Methodologically, it will be relevant to find out if they interfere with the reliability of the immunoassay by abolishing parallelism (Hersey et al., 2022; Khalil et al., 2018).
2. Tissue specificity. The assumption on which this review is based is that Nf are 100% specific for the neuronal and axonal compartment. This is not entirely true. Very small quantities of Nf can also be found in blood cells (erythrocytes, T lymphocytes), podocytes, testis, oocytes, cancerous tissue, stem cells and thymus. Quantitatively this may not matter as the Nf concentration measured from 50 neurons is more than what can be measured for 56.39 kg of CD34+ cells or erythrocytes (Petzold, Mondria, et al., 2010; Petzold, Tisdall, et al., 2011). Yet, why do these cells employ Nf? Has this to do with structure and stability?
3. Cancerous cells also express Nf as part of their cytoskeleton. Nf are an epigenetic factor in oncology (Calmon et al., 2015; Hasan et al., 2021; Li et al., 2020). Why is this the case? Do Nf help cancer to improve resistance against treatment? Or does it increase the risk of cancer? Does this open novel treatment strategies in oncology? Likewise, is there evidence for Nf to contribute to transport and propagation of certain neuroinvasive viruses (Gajdusek, 1985; Zhang et al., 2019)?
4. The need for a reliable test for Nf as a human body fluid biomarker for neurodegeneration has, because of analytical advantages, narrowed the field down to small proteolytic fragments of NfL. This line of research does not permit investigating the relevance of aggregate formation and PTMs of the larger protein subunits. Nf aggregate formation occurs spontaneously with impaired Nf stoichiometry for example in stem cells (Chao et al., 2007), transgenic models (Didonna & Opal, 2019; Julien, 2001; Rebelo et al., 2016), human disease (Adiutori et al., 2018), taphonomy and archaeology (Petzold et al., 2020). Future research should consider investigating into more detail the role of PTMs given the link with autoimmunity and aggregate formation (Zecha et al., 2022). For example there must be a reason why NfH is the most extensively phosphorylated protein of the human body. Why are Nfs to a large part intrinsically unstructured? Why are some Nfs able to self‐assemble into polymers and form intra‐ and extracellular aggregates in pathology? How does the averaged Nf stoichiometry vary between different regions in the human nervous system?
5. Most studies draw conclusions on CNS involvement based on pathological blood Nf concentrations. This may not always be correct (see Figure 1). NfL, NfH and NfM are also expressed in the PNS. Future method development work should include INA and PRPH to permit for more accurate separation of CNS from PNS injury. This is of relevance, for example for the interpretation of athletics related to increase in Nf levels.
6. The relationship between Nf isoforms and axonal conduction velocity should be revisited. Is axonal conduction speed only amplified through increase in axonal diameter, or does axonal Nf stoichiometry and PTMs such as phosphorylation also play a role? And related to this, if Nf isoforms are relevant to speed of information processing in the neuronal network, are they also relevant to understanding neuronal network memory?
7. Quality control and normal values for Nf concentrations. In order to become suitable for clinical routine, there is a need to capitalise on the current enthusiasm and consolidate reproducibility. The facts are that there remains a poor level of agreement between laboratories for reporting absolute Nf concentrations, even if using the same immunoassay. This error is frequently reported in the form of a metric called the coefficient of variation (CV). The inter‐laboratory CV was as high as 59% in the first global validation study (Petzold, Altintas, et al., 2010). This has not yet sufficiently improved in smaller‐scale repeat validation studies to give confidence to divert from the consensus recommendation for Nf batch analysis in expert laboratories (Petzold, Altintas, et al., 2010). It will be important to participate in External Quality control schemes to help individual laboratories to improve and continue gaining trust from the wider medical community in this test. This includes for laboratories to be internally consistent about cutoff levels and normal ranges reported in the literature. As reviewed in the section on FTLD, there is also a need for Nf isoform protein standard curves to be calibrated logically. This will include a revision of the Nf isoform protein sources which presently come from a range of rodents, pigs and bovine sources. It will be of advantage for the study of human diseases using human Nf protein. Such an approach will also be of help to more systematically investigate the presence of autoantibodies mentioned above.
  Next, the need for human biofluid‐specific normative data on Nf levels cannot be underestimated. Age, BMI and neurological comorbidities are all relevant. Likewise, a standardised approach to interpretation of these values is required. Absolute values, percentiles and Z‐scores are all used and each approach has advantages and limitations. On a group level, there remains a large overlap between health and disease. Overcoming these issues will pave the way for application of Nf to individualised, personal medicine.
8. The causal relationship of Nf body fluid levels with age should be investigated in more detail (Paris et al., 2022). The challenge is to separate physiological ageing from accelerated neurodegeneration. There are at least two approaches to this. One approach will be driven clinically by identifying relevant associations, comorbidities and lifestyle which drive age‐related neurodegeneration. The other approach will be motivated neurochemically by investigating Nf stoichiometry, PTMs, autoantibodies, proteolytic breakdown products and aggregates in order to better understand how the human body attempts on a molecular level to adapt to ageing.
9. There is a need for post‐mortem studies to better understand the distribution of Nf isoforms, PTMs, proteolytic breakdown products and aggregates in the human body. Such studies should include analysis of the reticuloendothelial system as likely relevant to the turn‐over of Nf isoforms in the human body. Such studies will also permit shedding more light on the previous point on the relationship of Nf body fluid concentrations with physiological ageing and pathology. For example are the differences in the Nf proteome found in the spleen of an individual who died from ALS compared to old age? Regarding the human brain, much more detailed region‐specific characterisation of Nf isoforms will be required (Budelier et al., 2022; Magliozzi et al., 2022; Sjölin et al., 2022). Expanding from post‐mortem studies to archaeology, Nf can contribute to investigating the evolutionary descent of the nervous system (Lasek et al., 1985; Petzold et al., 2020; Zalc & Colman, 2000).
10. Finally, there is an open field for biomarker discovery beyond Nf. Having been validated, Nf can be used as a guide for this line of research. Human body fluid samples with high Nf isoform concentrations taken in an acute clinical event can be selected for in‐depth analytical search of other biomarkers. Data derived from such experiments can be matched from the biomarker discovery profiles found in samples taken in chronic conditions, exacerbation of relapsing conditions, after treatment, from different age groups and from normal control individuals. In conclusion, samples with high Nf isoform concentrations also contain traces of other biomarkers helpful to further investigate neurodegeneration.

## 15 CONCLUSIONS

It can be concluded that Nf have become the first non‐disease‐specific biomarkers for neurodegeneration which can be quantified on an industrial scale from blood samples and many other body fluids. Consequently, there has been an exponential growth of Nf studies covering all medical specialities. Because Nf are such a sensitive biomarker for neurodegeneration they should not be used as a first‐line diagnostic test for a specific disease. Instead, they provide valuable information through the longitudinal dynamics of Nf concentrations on the background of a well‐defined clinical scenario. The interpretation of Nf concentrations will require adjusted normal ranges for age. Constitutional factors such as obesity, endocrinological problems such as diabetes mellitus, nephrological issues such as renal function and neurological comorbidities will also need to be considered. There is important information for prediction of disease severity and outcome with higher Nf concentrations indicating a poorer prognosis. Nf body fluid levels can increase prior to clinical onset of disease in individuals which harbour pathogenic mutations. The successful use of Nf as a secondary endpoint in several treatment trials already started to influence clinical trial design. In this context, batch analysis of Nf in experienced laboratories is strongly recommended. Finally, for patient safety, unexpected rise of Nf concentrations can alert clinicians to potential neurotoxic complications of interventions.

### AUTHOR CONTRIBUTION

This review was conceived, researched and written by Axel Petzold.

### CONFLICT OF INTEREST

The author declares no conflict of interest.

### OPEN RESEARCH BADGES

This article has been awarded Open Materials Badge for making the components of the research methodology needed to reproduce the reported procedure and analysis publicly available in the Supplements. More information about the Open Science Badges can be found at Open Science Framework

## Supporting information


Supplementary material S1

## Data Availability

Data are shared as Supplementary Material.

## Endnotes

^1^
The 2022 meta‐analysis (Zhang et al., 2022) did not refer to the 2019 meta‐analysis (Forgrave et al., 2019).

^2^
There was no response from the authors on the progress of this meta‐analysis when contacted by email on MAY 03 2022.

^3^
This meta‐analysis was submitted to the Journal on Jul 14 2021, but did not refer to the 2‐year earlier meta‐analysis which was more complete (Forgrave et al., 2019).

^4^
The 2019 meta‐analysis (Martin et al., 2019) did not refer to the 2018 meta‐analysis (Cai & Huang, 2018).

^5^
The 2021 meta‐analysis (Mondello et al., 2021) did not refer to the 2020 meta‐analysis (Gao et al., 2020b).
